# Intensified atomic utilization efficiency of single-atom catalysts for nitrate conversion via electrified nanoporous membrane

**DOI:** 10.1126/sciadv.ads6943

**Published:** 2025-07-09

**Authors:** Xiaoxiong Wang, Lea R. Winter, Xuanhao Wu, Yingzheng Fan, Yumeng Zhao, Jae-Hong Kim, Menachem Elimelech

**Affiliations:** ^1^ Institute for Ocean Engineering & Center of Double Helix, Tsinghua Shenzhen International Graduate School, Tsinghua University, Shenzhen 518055, China.; ^2^ Department of Chemical and Environmental Engineering, Yale University, New Haven, CT 06520, USA.; ^3^ Department of Environmental Engineering, Zhejiang University, Hangzhou 310058, China.; ^4^ State Key Laboratory of Urban Water Resource and Environment, Harbin Institute of Technology, Harbin 150090, China.; ^5^ Department Civil and Environmental Engineering, Department of Chemical and Biomolecular Engineering, Rice University, Houston, TX 77251-1892, USA.

## Abstract

Conventional electrochemical reactors for nitrate reduction typically suffer from limited reaction efficiency when applied for real-world water treatment due to poor utilization of electrocatalytic active sites. Here, we applied nanoporous electrofiltration to intensify atomic utilization by incorporating single-atom catalysts into an electrified membrane for reducing low-concentration nitrate to ammonia under realistic water conditions. We enhance the exposure of single atoms in nanopores by coating the catalysts on a carbon nanotube–interwoven membrane framework. Electrofiltration intensifies the transport and adsorption of nitrate in confined nanopores with highly exposed single-atom active sites to enhance reduction. The membrane enables a superior ammonia turnover frequency of 15.1 grams of nitrogen per gram of metal per hour, up to four orders of magnitude higher than that reported in the literature, under both high removal efficiency and Faradaic efficiency of over 86% when treating influents with a low nitrate concentration of 100 milligrams of nitrogen per liter in a residence time on the order of seconds.

## Teaser

Nanoporous electrofiltration intensifies atomic utilization of single-atom catalysts for low-concentration nitrate conversion.

## INTRODUCTION

Nitrate contamination threatens water resources and aquatic ecosystems (*1*, *2*). The excessive release of nitrate causes eutrophication and elevates reactive nitrogen concentrations in drinking water sources to potentially hazardous levels (*3*, *4*). The standard limit for nitrate in drinking water and groundwater is 10 to 20 mg of N liter^−1^ in countries such as the United States (*5*), the European Union (*6*), and China (*7*), but many water sources have exceeded this limit. On the other hand, the release of nitrate-containing waste streams to the environment represents a resource loss (*8*), where reactive nitrogen loss to the environment is estimated to reach 132 Tg of N year^−1^ in 2030 (*9*). Electrochemical reduction of nitrate to ammonia provides a potentially promising approach for simultaneously addressing nitrate contamination in water while recovering reactive nitrogen as ammonia under mild operating conditions and without external chemical input (*10*, *11*). In particular, nitrate reduction could offer a sustainable alternative to offset the fossil fuel–intensive Haber-Bosch process for synthesizing ammonia as fossil-free fertilizer or carbon-neutral fuel (*12*).

Most studies focus on recovering ammonia from wastewaters with high nitrate concentrations (*13*). However, nitrate concentrations in water and wastewater are typically below ~1 g of N liter^−1^ under the majority of conditions (*14*, *15*). When applied under realistic water conditions, electrochemical reduction of nitrate often exhibits limited reaction rate, Faradaic efficiency, and removal efficiency due to low reactant concentrations and competing reactions (*16*, *17*). Further, the reliance on relatively high loadings of metal catalysts raises concerns related to high treatment costs and hazardous metal release during long-term operation (*18*).

Single-atom catalysts (SACs) offer immense potential for minimizing the use of metals in electrochemical nitrate reduction, owing to their theoretical maximal utilization of metal atoms, uniform active sites, and unsaturated coordination environment (*19*). Iron, the most earth-abundant transition metal, has been identified as having desirable electron-donating capabilities and electrocatalytic activity toward nitrate-to-ammonia conversion in single-atom form (*20*, *21*). Single atoms anchored on defective N-doped carbon (NC) could further induce asymmetric charge distribution to optimize the adsorption of the intermediates on the active sites to intensify nitrate reduction (*22*, *23*). However, the metal loading of single atoms in catalysts is generally low (0.5 to 2.0 wt %) due to the high surface energy (*24*). The advantages of the single-atom active sites may not be fully realized under this limited loading when converting nitrate with low concentrations. This is because the formation of a thick diffusion boundary layer and electrostatic repulsion at the cathode interface hinder the mass transport and adsorption of the small number of nitrate molecules (*25*). Considering the difficulties of substantially increasing the loading of single atoms (*26*), investigating effective methods to intensify the atomic utilization efficiency is imperative for low-concentration nitrate conversion.

Applying flow-through electrofiltration may provide a potential alternative to enhance atomic utilization efficiency by improving the interaction between the low-concentration reactants and the single-atom active sites. Advection through the porous electrodes with high intrapore surface area can decrease the thickness of the diffusion boundary layer to a length scale of the pore radius (*27*). This indicates that the collision frequency between the reactants and the surface within the pores, especially in nanoscale pores, can be increased significantly. Thus, the utilization efficiency could be maximized if the SACs are functionalized uniformly inside the nanoscale pores. The enhanced exposure of the nitrate molecules to the surface active sites during transport through the small pores may further address the barriers associated with adsorption under electrostatic repulsion (*28*). However, limited methods are available for incorporating SACs with tailored structures in the nanopores of flow-through electrodes for water treatment. The effectiveness of nanoporous electrofiltration in improving the utilization efficiency of the SACs has also not been explored.

In this study, we apply nanoporous electrofiltration to intensify the atomic utilization by incorporating SACs into a reactive electrified membrane (EM) for nitrate-to-ammonia reduction at realistic conditions. We propose a facile EM fabrication method of coating a carbon nanotube (CNT)–interwoven framework with SACs to ensure sufficient exposure of the catalysts in the membrane nanopores. The free-standing CNT EM incorporating Fe single atom (Fe_1_)–anchored defective NC black (Fe_1_/NCB_d_@CNT-FEM) achieves both high removal efficiency and Faradaic efficiency of over 86% for reducing nitrate with a low concentration of 100 mg of N liter^−1^ to ammonia in a single-pass electrofiltration. Notably, the ammonia turnover frequency reaches an extremely high level of 15.1 g of N g of metal^−1^ hour^−1^ (60.1 mol of NH_3_ mol of Fe^−1^ hour^−1^), up to four orders of magnitude higher than that reported in the literature, under an Fe loading of only 0.65 wt % in the catalysts. We reveal the mechanisms involved in the enrichment of nitrate molecules within the confined nanopores for promoting nitrate transport, adsorption, and reduction by the highly exposed Fe_1_ active sites. We further demonstrate the viability of applying the EM to reduce nitrate at near-realistic conditions, and we propose a sequential electrofiltration method to ensure the near-complete removal of nitrate under different water conditions.

## RESULTS

### Fabrication and characterization of Fe_1_/NCB_d_@CNT-FEM

**Fig. 1.**
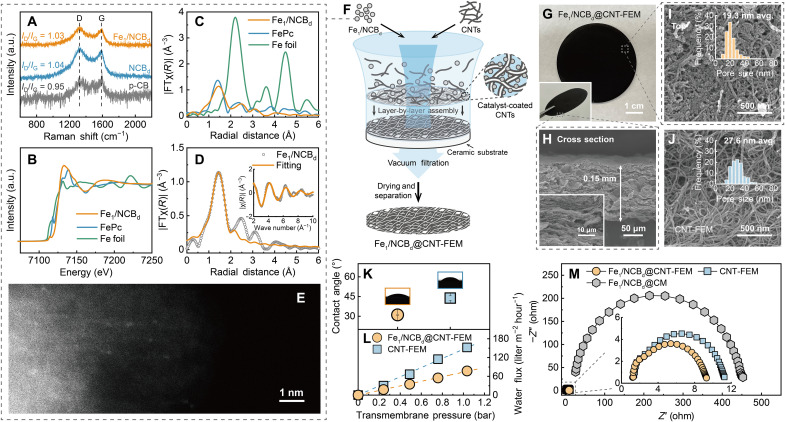
Fabrication and characterization of Fe_1_/NCB_d_@CNT-FEM. (**A**) Raman spectra of Fe_1_/NCB_d_, NCB_d_, and p-CB. a.u., arbitrary units. (**B**) Normalized Fe *K*-edge XANES and (**C**) FT-EXAFS spectra of Fe_1_/NCB_d_, iron phthalocyanine (FePc; Fe-N reference), and iron foil (Fe-Fe reference). (**D**) Fitting of the Fe_1_/NCB_d_ FT-EXAFS spectrum. Inset is the corresponding *K*-space spectrum. (**E**) High-angle annular dark-field scanning transmission electron microscopy (HAADF-STEM) image of Fe_1_ on NCB_d_. (**F**) Schematic illustrating the fabrication procedure of the Fe_1_/NCB_d_@CNT-FEM. (**G**) Photographs of the Fe_1_/NCB_d_@CNT-FEM. The inset indicates the free-standing nature of the membrane. (**H**) SEM images of the cross-sectional view of the Fe_1_/NCB_d_@CNT-FEM at low and high magnifications (inset). SEM images of the top view of (**I**) the Fe_1_/NCB_d_@CNT-FEM and (**J**) a free-standing electrified CNT membrane (CNT-FEM). The insets show the distribution and average value of the membrane pore sizes. (**K**) Water contact angles of the Fe_1_/NCB_d_@CNT-FEM and CNT-FEM. (**L**) Water flux of the Fe_1_/NCB_d_@CNT-FEM and CNT-FEM. The water permeabilities of the membranes, indicated by the fitted slopes, are 73.4 and 146.5 liters m^−2^ hour^−1^ bar^−1^ for the Fe_1_/NCB_d_@CNT-FEM and CNT-FEM, respectively. (**M**) Electrochemical impedance spectroscopy (EIS) spectra of the Fe_1_/NCB_d_@CNT-FEM, the CNT-FEM, and an Fe_1_/NCB_d_-functionalized ceramic membrane (Fe_1_/NCB_d_@CM) over a frequency range of 1 to 10^6^ Hz in 50 mM Na_2_SO_4_ solution.

We use a ligand-mediated method using 1,10-phenanthroline (1,10-phen) as a chelator for Fe_1_-anchored defective NC black (Fe_1_/NCB_d_) synthesis to offer a nitrogen coordination environment for isolating each Fe_1_ in the carbon black substrate, as shown in fig. S1 [scanning electron microscopy (SEM) image of the Fe_1_/NCB_d_ shown in fig. S2]. Inductively coupled plasma mass spectrometry (ICP-MS) analysis confirms the successful loading of Fe at 0.65 wt %. The N 1s spectrum obtained using x-ray photoelectron spectroscopy reveals different N species contained in the Fe_1_/NCB_d_, including pyridinic N, pyrrolic N, and graphitic N (fig. S3 and table S1). The Fe 1s spectrum shows peaks for Fe 2p_3/2_ and 2p_1/2_ with the absence of a peak for Fe^0^ at 706.5 eV. The intensity ratios between the D and G peaks (*I*_D_/*I*_G_) of the Fe_1_/NCB_d_ and NCB_d_ in the Raman spectra are 1.03 and 1.04, respectively, higher than that of pristine carbon black (p-CB; 0.95) (Fig. 1A). These results suggest a higher level of surface defects after nitrogen doping by introducing 1,10-phen. Heterogeneous nitrogen incorporation into a graphitic plane is known to facilitate the formation of point defects with lowered formation energy (*29*).

The x-ray absorption near-edge structure (XANES) spectra show higher edge energy of the Fe_1_/NCB_d_ compared with that of the Fe foil reference, demonstrating the positive oxidation state of Fe (Fig. 1B). Fourier-transformed extended x-ray absorption fine structure (FT-EXAFS) analysis suggests that the Fe atoms in the Fe_1_/NCB_d_ are primarily not in metallic Fe nanoparticles but in an atomically dispersed state (Fig. 1C). X-ray diffraction results are consistent with the absence of Fe metallic nanoclusters/nanoparticles, as the pattern obtained for Fe_1_/NCB_d_ is identical to that of NCB_d_ (fig. S4). The spectrum of Fe_1_/NCB_d_ is similar to that of the standard reference of iron phthalocyanine (FePc) for Fe-N coordination. In the FePc reference, the first strong peak at 1.42 Å is due to backscattering from four N atoms surrounding the Fe atom, while the second peak at 2.36 Å and the shoulder at 2.97 Å are due to backscattering from the eight C atoms of the pyrrole ring bonded to the N atoms and the four bridging N atoms, respectively (*30*). The first coordination shell peak (1.44 Å) of the Fe_1_/NCB_d_ is close to the Fe-N reference peak of FePc (1.42 Å) but differs from the Fe-Fe reference peak of Fe foil (2.24 Å). This suggests that the Fe atoms in the Fe_1_/NCB_d_ are coordinated with N atoms instead of with other Fe atoms. Fitting of the first coordination shell peak of Fe_1_/NCB_d_ FT-EXAFS spectrum shows an average Fe-N coordination number of 3.7 ± 0.1 with Fe-N radial distance at 1.99 Å, suggesting that the Fe atom is first coordinated with approximately four N atoms (Fig. 1D and table S2). The appearance of the second and the third peaks is due to backscattering from the surrounding C and N atoms beyond the first coordination, similar to the case in the FePc reference. High-angle annular dark-field scanning transmission electron microscopy (HAADF-STEM) provides visual evidence of the atomically dispersed Fe_1_ with the absence of nanoparticles (Fig. 1E).

We incorporate the Fe_1_/NCB_d_ into a nanoporous EM by coating a CNT-interwoven framework with the catalysts (Fig. 1F). Briefly, the same mass amount of Fe_1_/NCB_d_ and CNTs is blended in *N*,*N*′-dimethylformamide (DMF) containing 0.1 wt % polyacrylonitrile (PAN) as the binder using probe sonication. The Fe_1_/NCB_d_@CNT-FEM is fabricated by layer-by-layer assembly through vacuum filtration onto a ceramic substrate, followed by peeling off the monolithic carbonaceous layer from the substrate after rinsing and drying. The obtained free-standing EM is uniform and mechanically robust with an effective membrane area of 12.6 cm^2^ (Fig. 1G). The cross-sectional SEM images show a lamellar structure of the membrane with thickness of 0.15 mm (Fig. 1H). The comparison of the top-view SEM images between the Fe_1_/NCB_d_@CNT-FEM (Fig. 1I) and a free-standing unmodified CNT EM (CNT-FEM; Fig. 1J) indicates that the CNT-interwoven framework of the Fe_1_/NCB_d_@CNT-FEM is coated uniformly with the nanosized Fe_1_/NCB_d_ and the average membrane pore size reduces from 27.6 to 19.3 nm. This coating structure ensures the sufficient exposure of the catalysts during electrofiltration.

The incorporation of the Fe_1_/NCB_d_ slightly improves the hydrophilicity of the Fe_1_/NCB_d_@CNT-FEM, resulting in a lower water contact angle (31.0°) compared with the unmodified CNT-FEM (Fig. 1K). Further, the Fe_1_/N4CB_d_@CNT-FEM exhibits a 50% reduction in water permeability (73.4 liters m^−2^ hour^−1^ bar^−1^) compared with the CNT-FEM (146.5 liters m^−2^ hour^−1^ bar^−1^) (Fig. 1L). Thus, the coating structure results in a dense interwoven nature of the membrane with smaller pore size and lower water permeability. In addition, electrochemical impedance spectroscopy (EIS) reveals a ~50-fold difference in the amplitudes of the semicircles for both the Fe_1_/NCB_d_@CNT-FEM and CNT-FEM compared to an Fe_1_/NCB_d_-functionalized ceramic membrane (Fe_1_/NCB_d_@CM) (Fig. 1M). These results illustrate that the CNT framework is necessary to provide a sufficiently conductive substrate to enable efficient electron transfer reactions.

### Electrochemical nitrate reduction performance

**Fig. 2.**
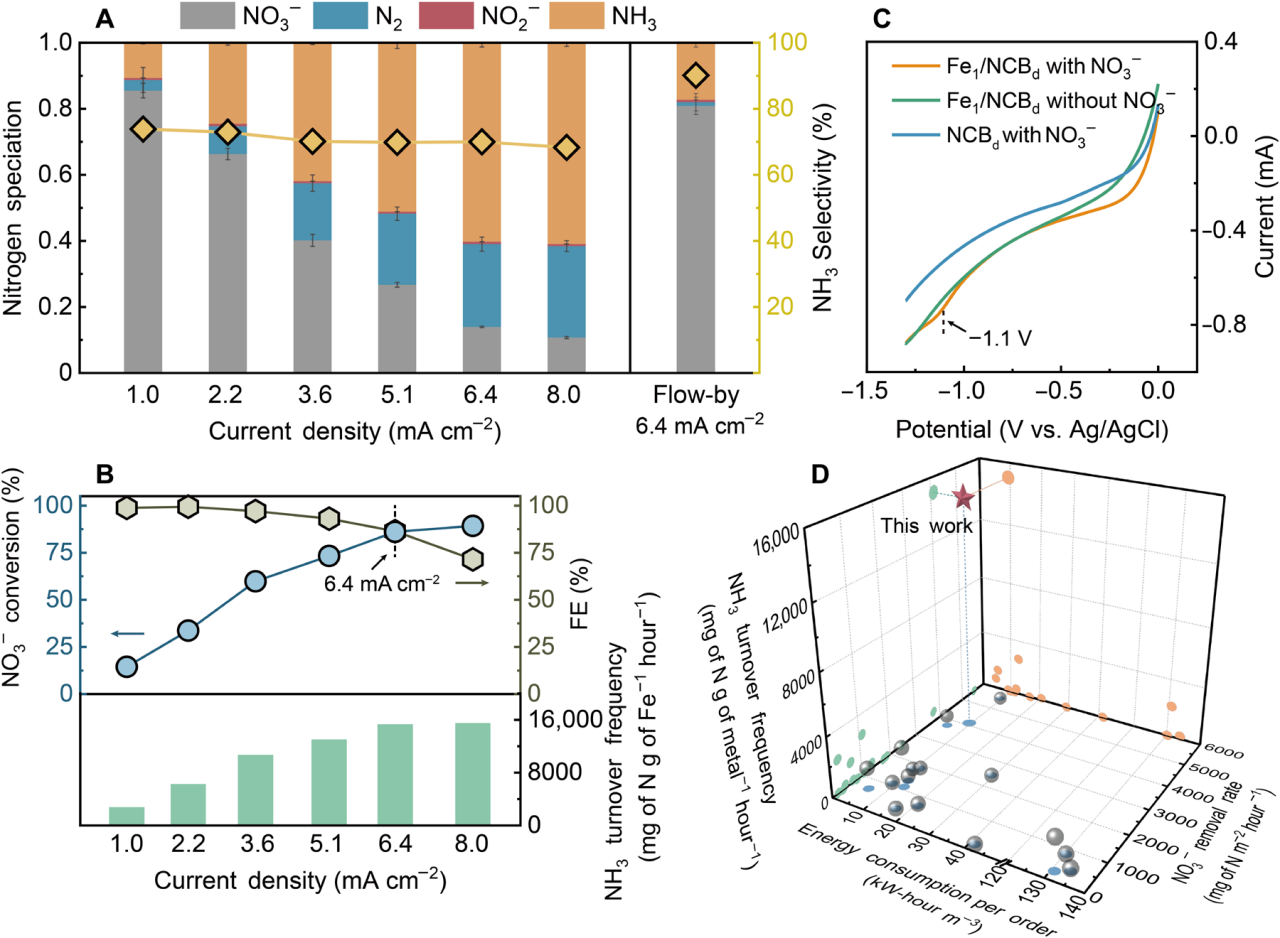
Electrochemical nitrate reduction performance using the Fe_1_/NCB_d_@CNT-FEM. (**A**) Effect of current density (1.0 to 8.0 mA cm^−2^) on nitrogen speciation in the permeate (left axis) and NH_3_ selectivity (right axis) using the Fe_1_/NCB_d_@CNT-FEM in the electrofiltration system. The right panel shows the results using an Fe_1_/NCB_d_-functionalized carbon paper electrode (catalyst loading of 1 mg of Fe cm^−2^) in a flow-by system at a current density of 6.4 mA cm^−2^ (electrode surface area of 12.6 cm^2^). Both filtration and flow-by experiments were performed by treating 60-ml feed solution containing NaNO_3_ (100 mg of N liter^−1^) and 50 mM Na_2_SO_4_ at neutral pH for 1 hour. (**B**) Changes of NO_3_^−^ conversion (left axis, top), Faradaic efficiency (FE) for NO_3_^−^ reduction (right axis, top), and NH_3_ turnover frequency (bottom) as a function of current density. (**C**) LSV curves of the Fe_1_/NCB_d_ and NCB_d_ in electrolytes without or with NO_3_^−^ addition [i.e., 50 mM Na_2_SO_4_ or 50 mM Na_2_SO_4_ plus NaNO_3_ (100 mg of N liter^−1^)] using glassy carbon as the support at a scan rate of 20 mV s^−1^. The dashed line indicates the potential where NO_3_^−^ reduction occurs. (**D**) Comparison of NH_3_ turnover frequency, NO_3_^−^ removal rate, and energy consumption per order of the Fe_1_/NCB_d_@CNT-FEM (red star) with other electrocatalysts reported in the recent studies (gray circles) with initial NO_3_^−^ concentration ranging from 50 to 100 mg of N liter^−1^ (table S3).

We evaluated the performance of the Fe_1_/NCB_d_@CNT-FEM by treating a feed solution containing a realistic nitrate (NO_3_^−^) concentration of 100 mg of N liter^−1^ at a neutral pH using a cross-flow electrofiltration system. As shown in Fig. 2 (A and B), the NO_3_^−^ conversion increases with current density, while the ammonia (NH_3_) selectivity remains relatively stable around 70%. Notably, the membrane is capable of reaching ~100% Faradaic efficiency for nitrate reduction when the current density is lower than 3.6 mA cm^−2^, although the removal of NO_3_^−^ is relatively low under these conditions. The Faradaic efficiency decreases slightly when further increasing the current density because competing reactions are more likely to consume the current in the presence of a low NO_3_^−^ concentration. We observe an optimal current density of 6.4 mA cm^−2^ for achieving both a high NO_3_^−^ removal efficiency of 86.1% and a high Faradaic efficiency of 86.4% in a single-pass filtration with a 10-s residence time in the membrane. The Fe_1_/NCB_d_@CNT-FEM reaches an NH_3_ production rate of 2.9 g of N m^−2^ hour^−1^ with an extremely high turnover frequency of 15.1 g of N g of metal^−1^ hour^−1^ (60.1 mol of NH_3_ mol of Fe^−1^ hour^−1^) under these conditions (fig. S5).

We performed flow-by experiments using the Fe_1_/NCB_d_@CNT-FEM (fig. S6) and an Fe_1_/NCB_d_-functionalized carbon paper electrode (Fig. 2A, right) to compare the NO_3_^−^ reduction performance between flow-through and flow-by modes. The NO_3_^−^ removal efficiency of both electrodes is below 20% at the same current density of 6.4 mA cm^−2^, primarily due to the limited mass transport and ineffective interaction of the low-concentration NO_3_^−^ with the catalysts. The role of nanoporous electrofiltration in enhancing mass transport and improving the utilization efficiency of Fe_1_ is further demonstrated by modifying the pore size of the EM. Under the same loading of the Fe_1_/NCB_d_, the turnover frequency increases by over 20% when the pore area is reduced by half (pore size reduced from 24 to 17 nm; fig. S7). In addition, the turnover frequency of the Fe_1_/NCB_d_@CNT-FEM is also higher than that of a membrane fabricated by trapping micrometer-sized Fe_1_-anchored NC (Fe_1_/NC) catalysts in the CNT-interwoven framework under the same Fe loading (fig. S8). These results suggest that electrified flow-through operation can overcome the limitations induced by diffusive transport, therefore maximizing the utilization of the highly exposed Fe_1_ in the nanopores of the membrane for nitrate reduction during electrofiltration.

We confirm that the activity for electrochemical NO_3_^−^ reduction is primarily due to the Fe_1_. A distinct peak at −1.1 V versus Ag/AgCl is observed only in the linear sweep voltammetry (LSV) curve of the Fe_1_/NCB_d_ in an electrolyte containing NO_3_^−^ (Fig. 2C). In addition, the NO_3_^−^ removal efficiency and NH_3_ turnover frequency of the Fe_1_/NCB_d_@CNT-FEM are over 30% higher than those obtained using a CNT-FEM incorporating the same amount of Fe as nanoparticles (63.9% and 11.6 g of N g of metal^−1^ hour^−1^, respectively; fig. S9). The Fe_1_/NCB_d_@CNT-FEM also exhibits higher NO_3_^−^ removal efficiency compared with a CNT-FEM incorporating NCB_d_ without Fe_1_ doping or incorporating Fe_1_/NC without the introduction of defects (fig. S10). These results demonstrate that both the use of single-atom Fe_1_ and the introduction of defects improve the NO_3_^−^ reduction efficiency of the Fe_1_/NCB_d_@CNT-FEM during electrofiltration. In addition, similar NO_3_^−^ reduction performance was observed with or without adding *t*-BuOH as the radical quencher to the feed solution (fig. S11), suggesting a direct electron transfer pathway for NO_3_^−^ reduction rather than reduction via atomic hydrogen generated through hydrogen dissociation. Electrofiltration enables higher local pH within the membrane compared to the feed water to further suppress hydrogen evolution reaction (fig. S12).

As shown in Fig. 2D, we compare the NO_3_^−^ reduction performance of the Fe_1_/NCB_d_@CNT-FEM with other electrocatalysts reported in the literature (details in table S3). We note that studies that are not included in our comparison focus mostly on developing catalysts to produce NH_3_ without considering NO_3_^−^ removal; these catalysts may not be able to maintain high NH_3_ production when near-complete NO_3_^−^ removal from water is required (*31*). The Fe_1_/NCB_d_@CNT-FEM is capable of achieving a NO_3_^−^ removal rate of 4.1 g of N m^−2^ hour^−1^ under both high NO_3_^−^ removal efficiency and Faradaic efficiency with an energy consumption per order of 9.4 kW·hour m^−3^. These values are competitive for treating water containing realistic NO_3_^−^ concentrations (50 to 100 mg of N liter^−1^), especially when considering the high water treatment throughput capacity of 60 ml hour^−1^ (corresponding to a residence time of only 10 s). Notably, the NH_3_ turnover frequency of the Fe_1_ in the membrane (15.1 g of N g of metal^−1^ hour^−1^) is up to four orders of magnitude higher than that reported in the literature, even when subtracting the contribution of NCB_d_ to NH_3_ generation (fig. S13). (Defects themselves might also serve as the active sites for NO_3_^−^ reduction in addition to adjusting the charge distribution of the Fe_1_-centered active sites.) In sum, the EM incorporating SACs for flow-through operation could significantly intensify the properties of the metal atoms for achieving highly efficient NO_3_^−^ removal and NH_3_ production from water with realistic NO_3_^−^ concentrations.

### Mechanism of Fe_1_ activity intensification under nanoporous electrofiltration

**Fig. 3.**
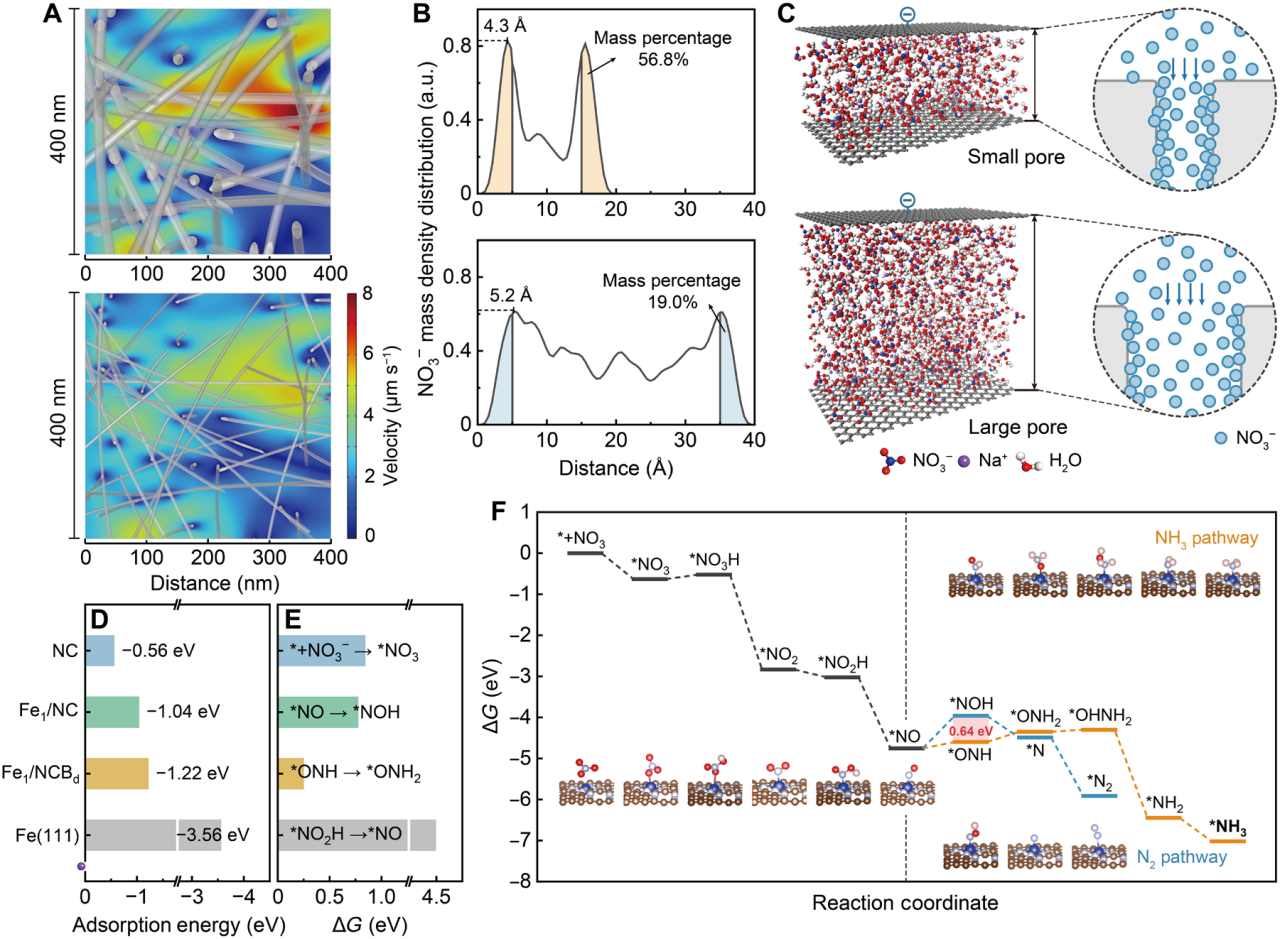
Theoretical analysis of electrochemical nitrate reduction using the Fe_1_/NCB_d_@CNT-FEM. (**A**) CFD simulating the velocity fields inside the membrane-interwoven frameworks with thick fibers (i.e., small membrane pores; top) and thin fibers (i.e., large membrane pores; bottom). (**B**) MD simulating mass density distributions of NO_3_^−^ between two parallel negatively charged graphene surfaces with vertical distances of 20 Å (representing small membrane pores) or 40 Å (representing large membrane pores). Areas under the curves for NO_3_^−^ distribution within a distance of 0.5 nm to the surfaces are filled. (**C**) MD simulation boxes describing the distribution of NO_3_^−^, Na^+^, and H_2_O molecules between surfaces or pores with different diameters (left) and corresponding schematics illustrating the distribution of NO_3_^−^ molecules when passing through small or large membrane pores (right). (**D**) Adsorption energies of NO_3_^−^ on NC, Fe_1_/NC, Fe_1_/NCB_d_, and Fe(111). (**E**) Energy barriers of the respective rate-determining steps for NO_3_^−^ reduction on NC, Fe_1_/NC, Fe_1_/NCB_d_, and Fe(111). (**F**) Gibbs free energy (Δ*G*) diagrams of the minimum energy pathways for electroreduction of NO_3_^−^ to NH_3_ (orange) and N_2_ (blue) by Fe_1_/NCB_d_. The structural models show different intermediates adsorbed on the Fe_1_/NCB_d_ surface during NO_3_^−^ reduction. Blue, brown, gray, red, and pink spheres represent Fe, C, N, O, and H atoms, respectively.

We first performed computational fluid dynamics (CFD) simulations to illustrate how flow-through electrofiltration intensifies mass transport to enhance the utilization of the catalysts in nanopores, as observed experimentally. The catalyst coating structure increases the nanofiber thickness while decreasing the pore size of the interwoven framework. Thus, simulation models were built considering water flow over interwoven frameworks with thick nanofibers (i.e., small membrane pores) and thin nanofibers (i.e., large membrane pores) and a flat plate (representing flow-by operation) with a constant NO_3_^−^ conversion rate at the exposed surfaces (fig. S14). The simulated velocity fields reveal that local water velocity is significantly intensified within the narrow channels of the nanofiber framework (Fig. 3A). This is crucial for maximizing the utilization of the highly exposed catalysts coated on the nanofibers. As a result, the NO_3_^−^ concentration decreases much faster with flow distance over the interwoven framework with thick nanofibers compared to the framework with thin nanofibers and the flat plate (fig. S15), enhancing NO_3_^−^ conversion (fig. S16). Therefore, coating SACs with small particle sizes on the interwoven framework might be more favorable to intensify atomic utilization compared with anchoring directly the single atoms on the framework.

We then used molecular dynamics (MD) simulations to investigate the effect of reducing the membrane pore size on the mass density distribution of NO_3_^−^ in the nanopore. The density of NO_3_^−^ molecules was slightly greater close to the surfaces (i.e., within 0.5-nm distance) compared to the other regions closer to the centerline of the large pore (Fig. 3B, bottom). Notably, the density distribution near the surface compared to the areas further from the surface increases markedly to 56.8% when the pore radius is reduced by half (Fig. 3B, top). The peaks of the mass density in the small pore (4.3 Å) are also closer to the surface than those in the large pore (5.2 Å). These results are consistent with the observed experimental trends, where nitrate conversion increased with smaller pore size (fig. S7). In addition, the increase in surface electronegativity has little effect on the distribution of NO_3_^−^ (fig. S17). Therefore, these results demonstrate that the enhanced adsorption capacity of catalysts located in small nanopores under high mass transport conditions can potentially mitigate the electrostatic repulsion at the electrode interface, as illustrated by Fig. 3C.

We further applied density functional theory (DFT) simulations to compare the NO_3_^−^ adsorption and charge transfer properties of the Fe_1_/NCB_d_ with NC and Fe_1_/NC without added defects (Fe_1_/NC). The projected density of states for NO_3_^−^ adsorption on the surfaces containing Fe reveals large band overlaps compared to those without Fe (fig. S18). The introduction of N defects further shifts the position of the d-band center of the Fe_1_/NCB_d_ toward the Fermi level, suggesting an enhanced capacity for NO_3_^−^ adsorption. In addition, the Fe_1_ also contributes to reducing the bandgap of the catalysts, which can facilitate charge transfer. This can be demonstrated by the greater Bader charge transfer from the Fe_1_/NCB_d_ (0.825 *e*) compared with that from the NC and the Fe_1_/NC (0.412 and 0.713 *e*; fig. S19). As a result, the NO_3_^−^ adsorption energy of the Fe_1_/NCB_d_ (−1.22 eV) is higher than that of the NC and Fe_1_/NC (Fig. 3D), which is attributed to the influence of the asymmetric charge distribution induced by the N defects on modifying the electronic structure of the Fe-N_4_ active sites.

We estimate the energy barriers associated with the rate-determining step for NO_3_^−^ reduction to NH_3_ on the different catalyst surfaces in Fig. 3E. Anchoring Fe_1_ to NC lowers the Δ*G* for the reduction of NO_3_^−^ to nitric oxide (NO) to 0.24 eV (figs. S20 and S21). NO is considered to be the divergent center for the final product selectivity of NO_3_^−^ reduction. The introduction of N defects could regulate the interaction between *ONH and the active sites of Fe_1_/NCB_d_ to reduce the Δ*G* of *NO hydrogenation (fig. S22). NO_3_^−^ reduction to NH_3_ via the *ONH pathway shows an extremely low energy barrier of 0.25 eV on the Fe_1_/NCB_d_ surface, which could be easily overcome with moderate energy input. In addition, hydrogenation of *NO on the N site (i.e., *ONH pathway) exhibits a lower Δ*G* of 0.64 eV compared with that on the O site (i.e., *NOH pathway; Fig. 3F). This helps to suppress N_2_ formation through *N coupling, therefore improving the NH_3_ selectivity.

### Operation under near-realistic conditions

**Fig. 4.**
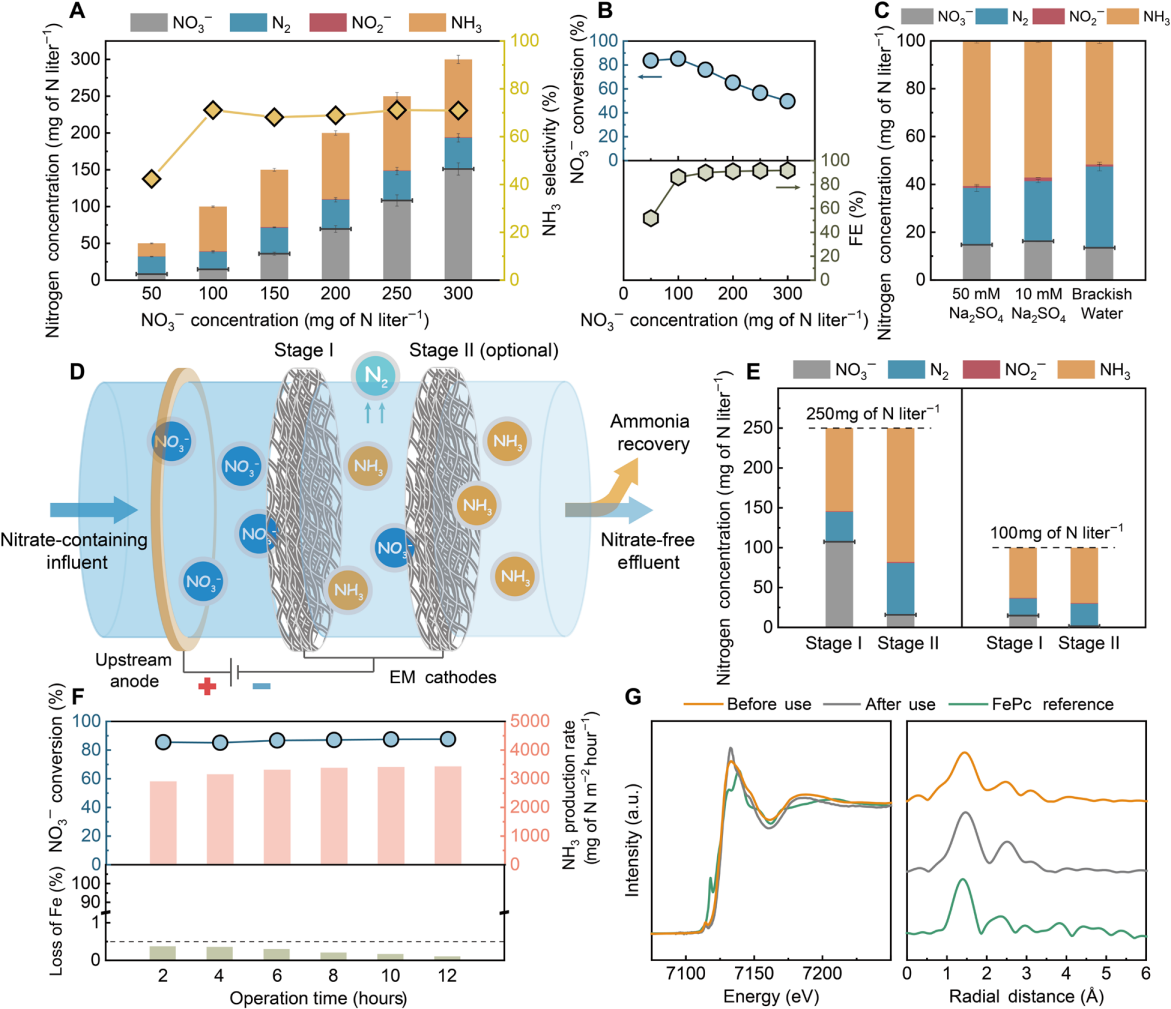
Electrochemical nitrate reduction under near-realistic conditions using the Fe_1_/NCB_d_@CNT-FEM. (**A**) Effect of initial NO_3_^−^ concentration (50 to 300 mg of N liter^−1^) on the distribution of nitrogen species (left axis) and NH_3_ selectivity (right axis) using the Fe_1_/NCB_d_@CNT-FEM. Black bars indicate the residual NO_3_^−^ concentration in the permeate. (**B**) NO_3_^−^ conversion (left axis, top) and Faradaic efficiency (right axis, bottom) as a function of initial NO_3_^−^ concentration. (**C**) NO_3_^−^ reduction performance of the Fe_1_/NCB_d_@CNT-FEM when treating different feed solutions containing NO_3_^−^ (100 mg of N liter^−1^) in 50 mM Na_2_SO_4_, 10 mM Na_2_SO_4_, or in simulated brackish water (constituents listed in table S4). (**D**) Schematic illustrating a sequential electrofiltration system for achieving near-complete NO_3_^−^ conversion. (**E**) NO_3_^−^ reduction performance of the sequential electrofiltration system using two Fe_1_/NCB_d_@CNT-FEMs for treating feed solutions with different initial NO_3_^−^ concentrations of 100 or 250 mg of N liter^−1^ in 50 mM Na_2_SO_4_ at neutral pH. (**F**) NO_3_^−^ conversion (left axis, top), NH_3_ production rate (right axis, top), and loss of Fe (bottom) of the Fe_1_/NCB_d_@CNT-FEM as a function of operation time for 12 hours. Experiments were performed using a feed solution containing NO_3_^−^ (100 mg of N liter^−1^) in 50 mM Na_2_SO_4_. (**G**) Normalized Fe *K*-edge XANES (left) and FT-EXAFS spectra (right) of the Fe_1_/NCB_d_@CNT-FEM before and after long-term operation. The constant current mode was applied at a fixed current density of 6.4 mA cm^−2^, unless otherwise noted.

The Fe_1_/NCB_d_@CNT-FEM exhibits superior NO_3_^−^ removal efficiency and NH_3_ turnover frequency. This is attributed to the high exposure of the catalysts with an asymmetric charge distribution of the Fe_1_ active sites in the confined membrane nanopores to facilitate the transport, adsorption, and conversion of the NO_3_^−^ molecules during flow-through electrofiltration. We further investigate the performance of the membrane for treating water that contains different concentrations of NO_3_^−^ and for electrolytes containing competitive ions. As shown in Fig. 4 (A and B), the Faradaic efficiency is maintained at ~90% with a relatively stable NH_3_ selectivity of ~70% when the initial NO_3_^−^ concentration is equal to or greater than 100 mg of N liter^−1^. The NO_3_^−^ conversion decreases with increasing initial concentration, although the rates of NO_3_^−^ removal and NH_3_ yield increase (figs. S24 and S25).

The NO_3_^−^ reduction efficiency for two-dimensional flow-by electrodes is generally sensitive to the electrolyte conditions. However, the Fe_1_/NCB_d_@CNT-FEM demonstrates similar NO_3_^−^ reduction performance when treating water with low ionic strength (10 mM Na_2_SO_4_) and simulated brackish water containing typical competitive ions (constituents listed in table S4) under flow-through operation compared to the more concentrated feed solution (50 mM Na_2_SO_4_), as shown in Fig. 4C. These results demonstrate the feasibility of using the membrane for removing low concentrations of NO_3_^−^ at near-realistic electrolyte conditions.

To reduce the residual NO_3_^−^ concentration in treated water following ammonia recovery, we propose a sequential electrofiltration method for realizing near-complete conversion of NO_3_^−^ under different concentrations, as illustrated in Fig. 4D. The free-standing property enables the membranes to be stacked for multistage NO_3_^−^ reduction. For example, the Fe_1_/NCB_d_@CNT-FEM can remove nearly 150 mg of N liter^−1^ of the NO_3_^−^ from a feed solution with an initial concentration of 250 mg of N liter^−1^ in a single-pass electrofiltration. Applying sequential electrofiltration using two membranes removes a total of 235 mg of N liter^−1^ NO_3_^−^ with a residual concentration lower than 15 mg of N liter^−1^ (Fig. 4E, left), which meets standards for total nitrogen discharge in most cases after separating the NH_3_ products. Further, NO_3_^−^ (100 mg of N liter^−1^) could be almost completely removed (98.5%) in a sequential electrofiltration, thus mitigating eutrophication at water discharge points (Fig. 4E, right).

The Fe_1_/NCB_d_@CNT-FEM maintains a stable NO_3_^−^ removal efficiency of ~85% and an NH_3_ production rate of 3 g of N m^−2^ hour^−1^ in a single-pass electrofiltration throughout long-term operation (Fig. 4F, top). The loss of Fe from the membrane is negligible (<0.4%; Fig. 4F, bottom). In addition, the white line intensities of the Fe *K*-edge XANES spectra for the membrane before and after the long-term operation are similar (Fig. 4G, left). The FT-EXAFS spectrum of the Fe_1_/NCB_d_ in the membrane after use is identical to the FePc reference, showing the Fe-N first coordination shell peak of Fe-N at 1.44 Å and lacking a peak related to Fe-Fe coordination (Fig. 4G, right). These results demonstrate the robustness of the catalytic structure during long-term NO_3_^−^ reduction.

## DISCUSSION

Developing high-throughput continuous-flow electrodes with minimal metal usage is crucial for decentralized nitrate removal and ammonia synthesis from real water. In this study, we demonstrate the feasibility of applying flow-through electrofiltration to intensify atomic utilization efficiency by functionalizing Fe SACs in the nanopores of a reactive EM for rapid nitrate reduction to ammonia at realistic conditions (fig. S26). Coating the CNT-interwoven framework with the SACs provides highly exposed Fe_1_ active sites within the small nanopores of the membrane. Flow-through electrofiltration further intensifies the transport and adsorption of the low-concentration nitrate in the confined nanopores to facilitate their conversion. The EM achieves over 86% nitrate removal with a high Faradaic efficiency of nearly 90% in a single-pass electrofiltration (residence time of only 10 s) when treating water influent with a relatively low initial concentration of 100 mg of N liter^−1^ at neutral pH. Notably, an extremely high ammonia turnover frequency of 15.1 g of N g of metal^−1^ hour^−1^ (60.1 mol of NH_3_ mol of Fe^−1^ hour^−1^) is obtained, which is up to four orders of magnitude higher than that reported in the literature. This results in an ammonia production capacity comparable to that of converting high-concentration nitrate through conventional flow-by systems. Thus, the application of nanoporous electrofiltration in flow-through operation may potentially address the challenges involved in electrified flow-by operation by maximizing the atomic utilization efficiency for nitrate removal and ammonia synthesis from a broad range of water and wastewater.

Our free-standing CNT-based membrane provides a versatile platform to incorporate SACs for real-world applications, considering its facile and potentially economical fabrication process. Further, the method based on pyrolyzing metal-organic complexes doped on an inexpensive carbon black substrate has been demonstrated to be scalable for synthesizing SACs on kilogram scales (*32*). The intensified atomic utilization during nanoporous electrofiltration offers a promise for applying practical-scale SAC-functionalized EMs for water treatment with high efficiency and low cost. We demonstrate the viability of applying our EM for treating water with low ionic strength and competitive ions, and we propose a sequential electrofiltration method for realizing near-complete nitrate conversion in water to meet the discharge standards after ammonia separation. The compact and modular construction also makes EMs suitable for decentralized water treatment applications. In addition, the local synthesis of ammonia from nitrate-containing water using EMs may enable direct utilization of the water for fertigation applications (*33*). The synthesized ammonia can also be separated for on-site fertilizer and energy production by integrating the EMs with a gas-stripping hydrophobic membrane for continuous-flow sequential electrofiltration (*34*, *35*). Therefore, this study provides a promising decentralized approach for near-complete nitrate removal and sustainable ammonia synthesis from real water for both eutrophication mitigation and fossil-free fertilizer or carbon-neutral fuel generation.

## MATERIALS AND METHODS

### Synthesis and characterization of Fe_1_/NCB_d_

Iron(III) chloride and 1,10-phen were dissolved in ethanol at a molar concentration ratio of 1:3. After mixing with carbon black powder, the suspension was shaken overnight and subsequently dried at 80°C. The obtained powder was then calcined using a tube furnace (STF1200, Across International) under Ar atmosphere at 600°C for 2 hours at a heating rate of 5°C min^−1^.

The Fe content in the Fe_1_/NCB_d_ was quantified by ICP-MS (ELAN DRC-e, PerkinElmer). Raman spectroscopy (LabRAM HR Evolution, Horiba) was carried out to observe the characteristic D and G bands of the catalysts. X-ray absorption spectroscopy at the Fe *K*-edge was measured at Beamline 8-ID of the National Synchrotron Light Source II at Brookhaven National Laboratory, using a Si (111) double-crystal monochromator and a passivated implanted planar silicon fluorescence detector. XANES data were collected at room temperature, with energy calibrated with an Fe foil. The morphology of Fe_1_/NCB_d_ was analyzed using aberration-corrected HAADF-STEM at 300 kV.

### Fabrication and characterization of Fe_1_/NCB_d_@CNT-FEM

Fifty milligrams of Fe_1_/NCB_d_ and 50 mg of pristine CNTs were dispersed in 40 ml of DMF containing 0.1 wt % PAN, followed by sonication for 20 min using an ultrasonic probe. We applied layer-by-layer assembly by vacuum filtering 2 ml of the as-prepared suspension onto a ceramic membrane substrate (0.45 μm; Sterlitech). The prepared membrane with 20 layers in total was then rinsed with deionized (DI) water, followed by drying at 90°C for 12 hours. The Fe_1_/NCB_d_@CNT-FEM was obtained by directly peeling the whole carbonaceous layer off of the ceramic membrane substrate after drying.

SEM (SU8230, Hitachi) was used to investigate the surface and cross-sectional morphologies of the EMs. Membrane pore size distribution was estimated by analyzing the surface SEM images using Nano Measurer software. Water contact angles were measured by the sessile drop method using a contact angle goniometer (OneAttension, Biolin Scientific). Water flux was calculated by dividing the permeate flow rate by the effective membrane area (i.e., 12.6 cm^2^). Electrochemical measurements were performed using an electrochemical workstation (CHI 660E, CH Instruments) in a typical three-electrode electrochemical cell containing the working electrode, a mixed metal oxide electrode as the counter electrode, and an Ag/AgCl electrode as the reference electrode. EIS of the EMs was conducted by applying frequencies ranging from 1 to 10^6^ Hz in a 50 mM Na_2_SO_4_ solution at open-circuit voltage. LSV curves of the Fe_1_/NCB_d_ and NCB_d_ were collected at a scan rate of 20 mV s^−1^ in 50 mM Na_2_SO_4_ solution with or without NaNO_3_ (100 mg of N liter^−1^) addition using glassy carbon as the support.

### Electrofiltration experiments

Electrofiltration experiments were performed using a cross-flow membrane filtration system (photographs; see fig. S27). The Fe_1_/NCB_d_@CNT-FEM and a mixed metal oxide mesh electrode were placed in an electrofiltration cell with 1-cm spacing, serving as the cathode and anode, respectively. Electrolytes containing 50 mM Na_2_SO_4_ with different concentrations of NaNO_3_ at a neutral pH of 7.5 were used as the feed solution. The feed was circulated at a flow rate of 200 ml min^−1^ by a peristaltic pump, and the permeate flow rate was controlled to 1 ml min^−1^, corresponding to a water residence time of 10 s. We performed sequential electrofiltration by stacking two of the free-standing EMs in the electrofiltration cell with a macroporous polyester fabric placed in between to separate the membranes. Unless otherwise noted, nitrogen speciation was quantified on the basis of both colorimetric methods and ion chromatography (930 Compact, Metrohm) after 1 hour of operation. All experiments were conducted in triplicate (*n* = 3), and data are presented as mean values ± SD. One-way analysis of variance with the Tukey’s post hoc test was performed to analyze significant differences (*P* < 0.05) using Origin 2022 software.

### Simulations

CFD simulation was executed to calculate the nitrate concentration distributions in the membrane using COMSOL Multiphysics software. A cube fluid domain was constructed with a side length of 400 nm. Structures of a flat plate or nanofiber-interwoven frameworks with different diameters of 12 nm (representing thick fibers) or 6 nm (representing thick fibers) were built in the domain. Convective velocity was computed based on the Navier-Stokes equations using laminar flow physics. The mass transport of nitrate was simulated using the diffusion equation, considering the concentrations meet the dilute species transfer interface under different pore conditions. The model reflected surface reactions and the overall reaction satisfies the Fick’s diffusion law. The inlet boundary was set to a flow rate of 2.4 μm s^−1^ and a surface reaction rate of 2 × 10^−4^ m s^−1^.

A double-layer graphite (001) surface was constructed to simulate the membrane pore channel in Material Studio software for MD simulations. Na^+^, NO_3_^−^, and H_2_O molecules were pressed between the double layers via the amorphous cell module with a molar ratio of 1:1:5. The Forcite module was applied for geometric optimization with the universal force field for the composite model. The convergence accuracies were 0.001 kcal mol^−1^ for energy, 0.5 kcal mol^−1^ Å^−1^ for force, and 0.015 Å for displacement, respectively. The maximum repetition number was set as 5000 to ensure the optimization of the structure. The cutoff radius of the van der Waals force was chosen to be 12 Å. The MD algorithm with the canonical ensemble was performed for geometrically optimized structures with a random initial velocity at a temperature of 298 K. A rate scale temperature control method was selected for minimal temperature amplitude. The total running time was 1000 ps, and statistical analysis of the properties was performed on the final structure.

Spin-polarized first-principles DFT calculations were performed using the Perdew-Burke-Ernzerhof exchange-correlation functional implemented in the Vienna Ab initio Simulation Package. The projector augmented wave method and the corresponding pseudopotentials were used. A cutoff energy of 500 eV was set for the plane wave basis and a Monkhorst-Pack *k*-point sampling of 2 by 2 by 1 was set for converging. The convergence thresholds for the energy and force were 10^−5^ eV and 0.02 eV Å^−1^, respectively. Grimme’s DFT-D3 correction method was included to describe the weak dispersion interactions during surface adsorption.

## Supplementary Materials

### This PDF file includes:

Supplementary Text Figs. S1 to S29 Tables S1 to S4 References
