# Supplementary material for: Intensified atomic utilization efficiency of single-atom catalysts for nitrate conversion via electrified nanoporous membrane
Source: Sci Adv. 2025 Jul 9;11(28):eads6943. doi: 10.1126/sciadv.ads6943 (PMC12239952; doi:10.1126/sciadv.ads6943)
Supplement: Supplementary file 1 — Supplementary Text Figs. S1 to S29 Tables S1 to S4 References [file sciadv.ads6943_sm.pdf]

Science Advances
AAAS

Supplementary Materials for

Intensified atomic utilization efficiency of single-atom catalysts for nitrate conversion via electrified nanoporous membrane

Xiaoxiong Wang *et al.*

Corresponding author: Xuanhao Wu, xuanhao.wu@zju.edu.cn; Jae-Hong Kim, jaehong.kim@yale.edu; Menachem Elimelech, menachem.elimelech@rice.edu

Sci. Adv. **11**, eads6943 (2025)
DOI: 10.1126/sciadv.ads6943

This PDF file includes:

Supplementary Text
Figs. S1 to S29
Tables S1 to S4
References

Supplementary Text

Chemicals and Materials

Iron (III) chloride ($FeCl_3$, ≥99.9%), 1,10-phenanthroline (1,10-phen, ≥99%), carbon nanotubes (CNT, multi-walled, OD × L: 6–13 nm × 2.5–20 μm, >98%), N,N-dimethylformamide (DMF, ≥99.8%), polyacrylonitrile (PAN), sodium sulfate (Na_2SO_4, ≥99.0%), *tert*-butanol (*t*-BuOH, ≥99.0%) were purchased from Sigma-Aldrich (St. Louis, MO, USA). Carbon black powder (CB, Emperor 2000) was purchased from Cabot Corporation (Boston, MA, USA). Hydrochloric acid (HCl, 36.5–38.0%) was purchased from J.T. Baker (Phillipsburg, NJ, USA). Argon gas (UHP, 100%) was purchased from Airgas (Sacramento, CA, USA). Deionized (DI) water was obtained from a Milli−Q system (Millipore, Billerica, MA, USA).

Acid Treatment of CNTs

The as-received CNTs were pre-treated in a concentrated HCl solution (36.5–38.0%) at 90 °C under refluxing for 12 h, followed by washing with DI water until neutral and drying at 60 °C for 12 h for preparing the pristine CNTs.

Synthesis of Fe_{NP}/CB and Fe_1/NC

Fe_{NP}/CB was synthesized by pyrolysis of Fe-doped carbon black at 600 °C under 5% H_2/Ar atmosphere. Fe_1/NC catalysts were synthesized by pyrolysis of Fe-doped ZIF-8 precursors at 900 °C under Ar atmosphere.

Fabrication of EMs for control experiments

Fe_1/NC@CNT-FEM, Fe_{NP}/CB@CNT-FEM, NCB_d@CNT-FEM, CNT-FEM, and Fe_1/NCB_d@CM were fabricated according to the fabrication method for Fe_1/NCB_d@CNT-FEM, as described in the Methods section, but replacing the Fe_1/NCB_d by Fe_1/NC or Fe_{NP}/CB with same Fe mass loading, replacing by NCB_d with same catalyst mass loading, without Fe_1/NCB_d addition, or without CNT addition and active layer separation. Membrane pore size was controlled by adding inactive pristine CB prior to the catalysts during the vacuum filtration procedure.

Additional methods for catalyst and membrane characterization

The morphology of the Fe_1/NCB_d was investigated using SEM (SU8230, Hitachi). XPS analysis was conducted using a PHI VersaProbe II Scanning XPS Microprobe system with monochromatic Al Kα radiation (1486.6 eV). X-ray diffraction (XRD, Rigaku SmartLab) analysis was performed using Cu Kα radiation (λ = 1.542 Å) at a scan rate of 5° min^{-1} within the 2θ range of 5–60°. Membrane pore volume was determined by the weight difference between a wet and dry membrane. The residence time of water within the membrane was calculated by dividing the pore volume by the permeate flow rate.

XAFS Data Fitting

Athena software was used for XAFS data processing, including conversion of raw data to μ(E) spectra, background subtraction and normalization, and Fourier transformation and plotting. Artemis software was used for the analysis of EXAFS data using theoretical standards, including setting the range of the Fourier transform from *K*-space (*K* weight = 3, *K* = 3–10) and fitting range parameters in *R*-space (*R* = 1–2.2 Å). Interatomic distance is the bond length between central atoms and surrounding coordination atoms. σ^2 is the Debye-Waller factor, standing for the thermal and static disorder in absorber–scattered distance.

Batch Setup

A batch system was applied to compare the electrochemical NO_3^- reduction performance between flow-by (batch) and flow-through (filtration) modes using the Fe_1/NCB_d@CNT-FEM. The batch system consisted of a reactor with the membrane and an MMO mesh electrode with a spacing of 1 cm. To maintain consistency with the conditions of the electrofiltration experiment, batch experiments were performed for the treatment of 60-mL feed solution for 1 h at a current density of 6.4 mA cm^{-2} and a string rate of 500 rpm.

Nitrogen speciation analysis

NO_3^-, NO_2^-, and NH_4^+ concentrations in both the permeate and the feed were quantified after 1 h of operation. The concentrations of NO_3^-, NO_2^-, and NH_4^+ were determined according to the cadmium reduction method, the diazotization method, and the Nessler method, respectively, using assay kits (HI93728 for NO_3^-, HI93707 for NO_2^-, and HI93715 for NH_4^+, Hanna Instruments). The standard curves for the colorimetric quantification are shown in Fig. S28. Ion chromatography (IC, 930 Compact, Metrohm) was also employed to double-check the concentrations of the nitrogen ions. N_2 content is calculated from the mass balance, considering that the formation of other N-species is negligible. Blank experiments were performed to confirm the accuracy of the nitrogen analysis methods used in this study (Fig. S29).

Faradaic efficiency

Faradaic efficiency of nitrate reduction was calculated as

$$FE_{nitrate} = \frac{z \times F \times J \times (C_0 - C)}{j} \tag{1}$$

where z is the electron transfer number of NO_3^- reduction, determined based on the nitrogen speciation, F is the Faraday constant (96485 C mol^{-1}), J is the water flux, j is the current density, and C_0 and C are the NO_3^- concentrations before and after treatment, respectively.

Evaluation on ammonia generation

1) Ammonia production rate ($P_{ammonia}$) was calculated using

$$P_{ammonia} = J \times C_{ammonia} \tag{2}$$

where $C_{ammonia}$ is the NH_3 concentration in the permeate.

2) Ammonia turnover frequency ($TOF_{ammonia}$) was calculated as

$$\mathrm{TOF}_{\mathrm{ammonia}} = \frac{C_{\mathrm{ammonia}} \times Q}{M} \tag{3}$$

where Q is the permeate flow rate and M is the mass of metal involved in NO_3^- reduction.

Electrical Energy Consumption

Energy consumption per order (E_{EO}) for NO_3^- reduction during electrofiltration was evaluated using

$$\mathrm{E}_{\mathrm{EO}} = \frac{U_{\mathrm{cell}} \times I}{Q \times \log\left[\frac{C_0}{C}\right]} \tag{4}$$

where U_{cell} is the applied voltage and I is the current.

Adsorption energy

Adsorption energy of different species on the surface was calculated by

$$E_{\mathrm{ads}} = E_{\mathrm{x/surface}} - E_{\mathrm{x}} - E_{\mathrm{surface}} \tag{5}$$

where $E_{x/surface}$, E_x, and $E_{surface}$ are the energies of the slab with adsorbed species, the adsorbed species in a cubic cell, and the energies of the slab, respectively.

Gibbs free energies

Adsorption Gibbs free energies (ΔG) were calculated through

$$\Delta G = \Delta E + \Delta E_{\mathrm{ZPE}} - T \times \Delta S \tag{6}$$

where ΔE is the reaction energy, ΔE_{ZPE} and ΔS are the changes in zero-point energy and entropy, respectively, between the adsorbed state and free state, which can be obtained from the vibrational frequencies and standard thermodynamic data, and T is the temperature.

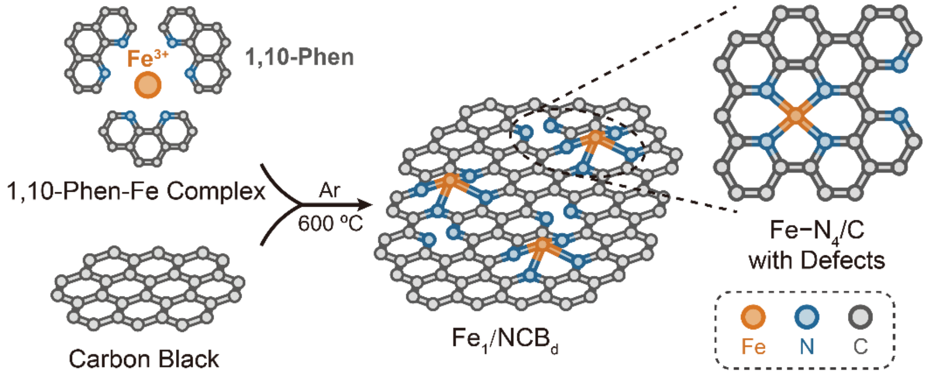

Fig. S1. Schematic illustrating the synthesis procedure of the Fe_1/NCB_d catalysts.

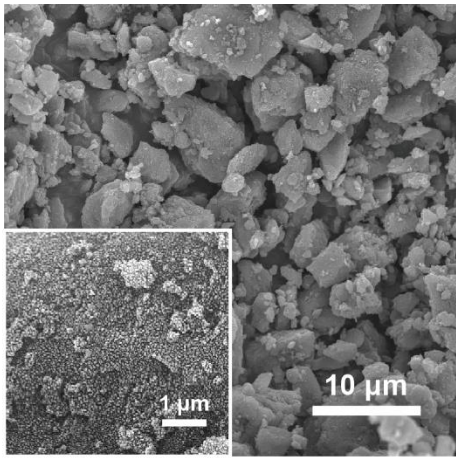

Fig. S2. SEM image of the Fe_1/NCB_d.

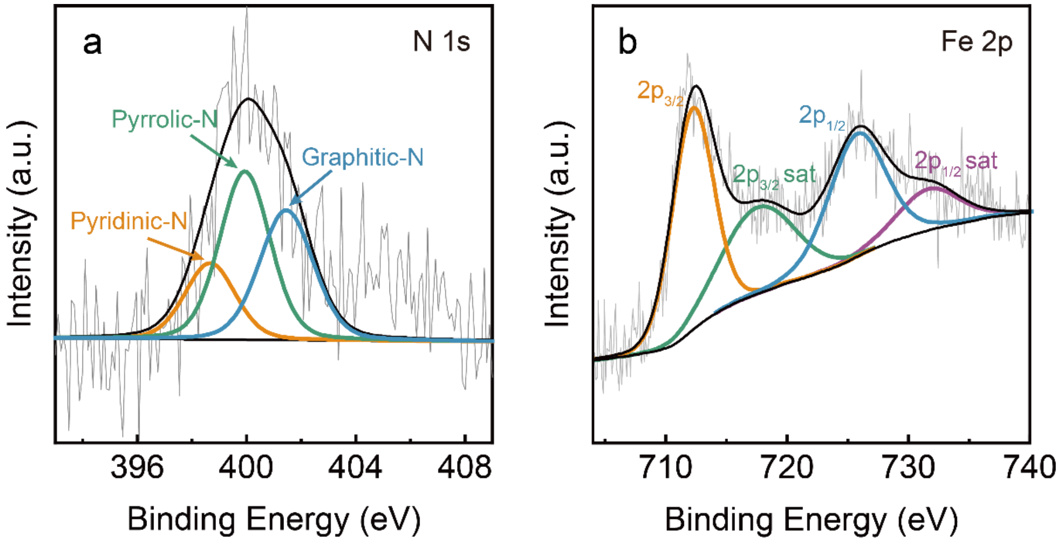

Fig. S3. XPS spectra of (a) nitrogen (N 1s) and (b) iron (Fe 2p) of Fe_1/NCB_d. Grey and black lines represent the spectral data and the curve-fitted spectra, respectively.

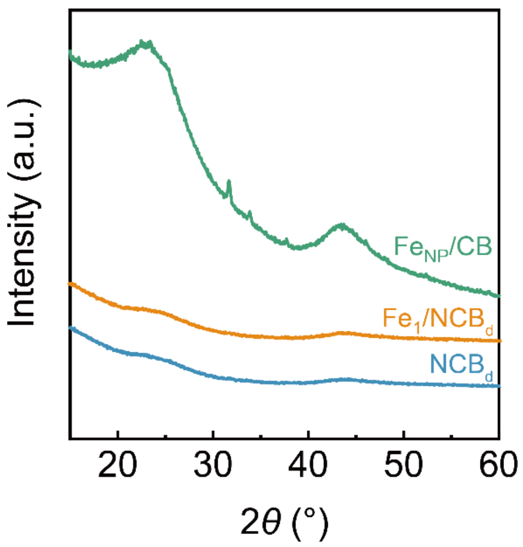

Fig. S4. XRD spectra of Fe$_{NP}$/CB, Fe$_1$/NCB$_d$, and NCB$_d$.

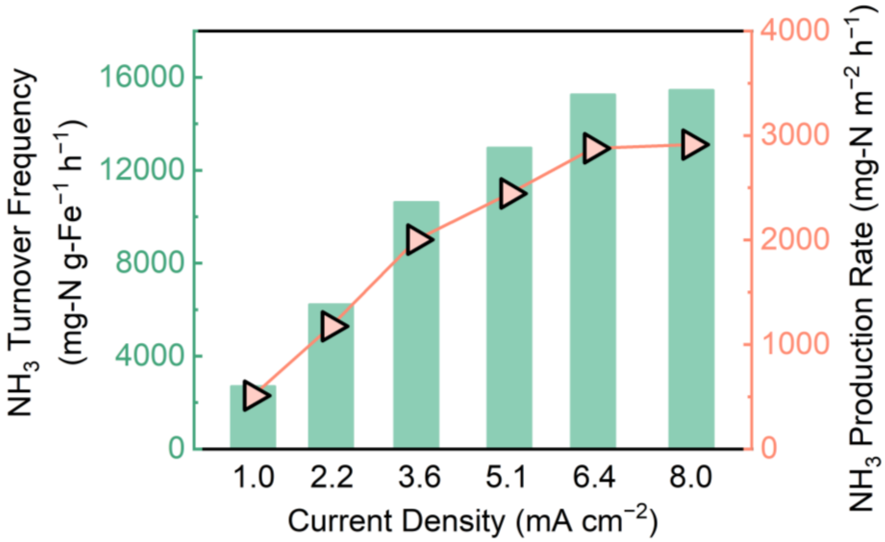

Fig. S5. Effect of current density (1.0–8.0 mA cm^{-2}) on NH_3 turnover frequency (left axis) and NH_3 production rate (right axis) for the Fe_1/NCB_d@CNT-FEM.

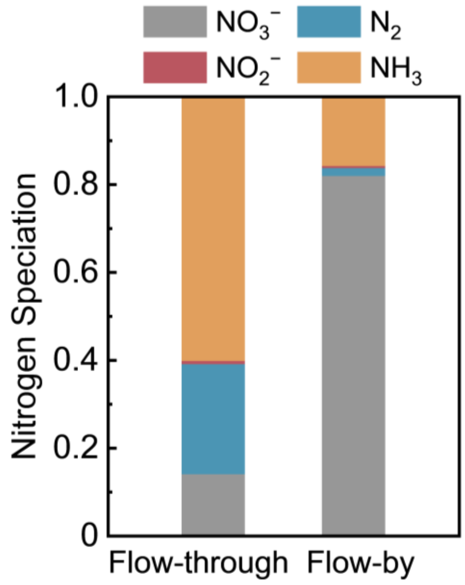

Fig. S6. Effect of flow-through and flow-by operation mode on nitrogen speciation in the permeate using the Fe_1/NCB_d@CNT-FEM at a current density of 6.4 mA cm^{-2}.

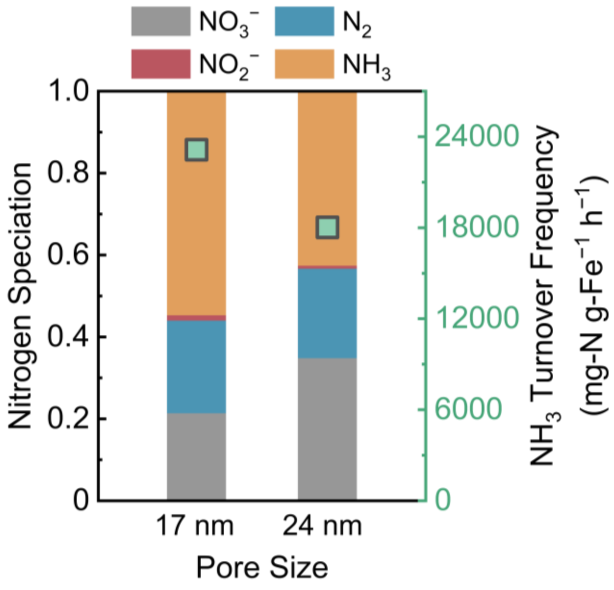

Fig. S7. Effect of membrane pore size on nitrogen speciation (left axis) in the permeate and NH_3 turnover efficiency (right axis). The amount of the Fe_1/NCB_d used for fabricating the membranes was reduced to 60% of the amount used in other tests (i.e., 30 mg) to obtain more distinct results.

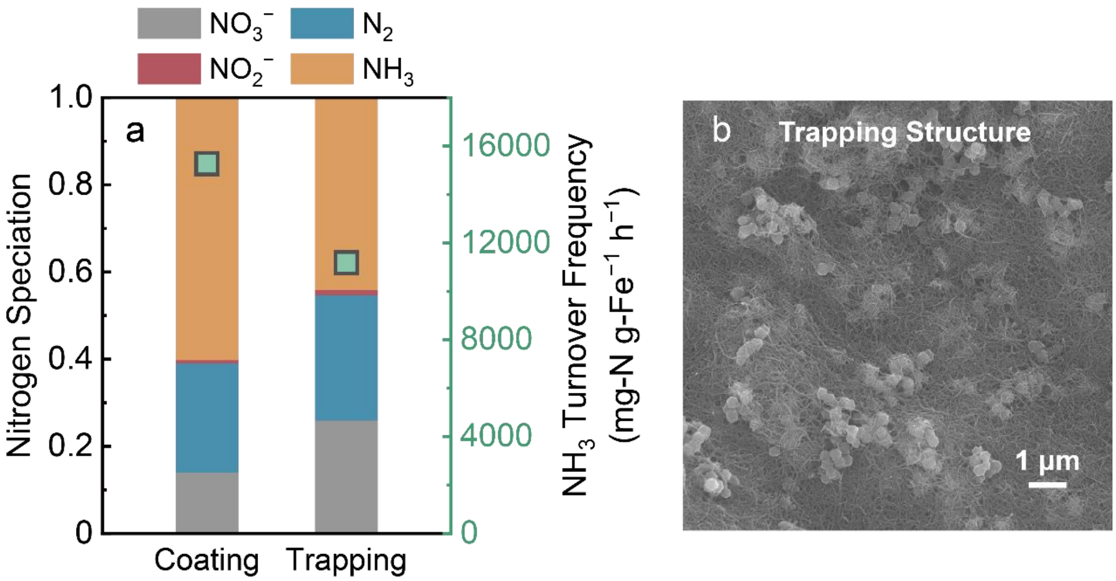

Fig. S8. (a) Effect of applying coating or trapping structure for incorporating Fe_1 anchored N-doped carbon catalysts in the CNT interwoven frameworks under the same Fe loading on nitrogen speciation (left axis) in the permeate and NH_3 turnover efficiency (right axis). (b) SEM image of the trapping structure.

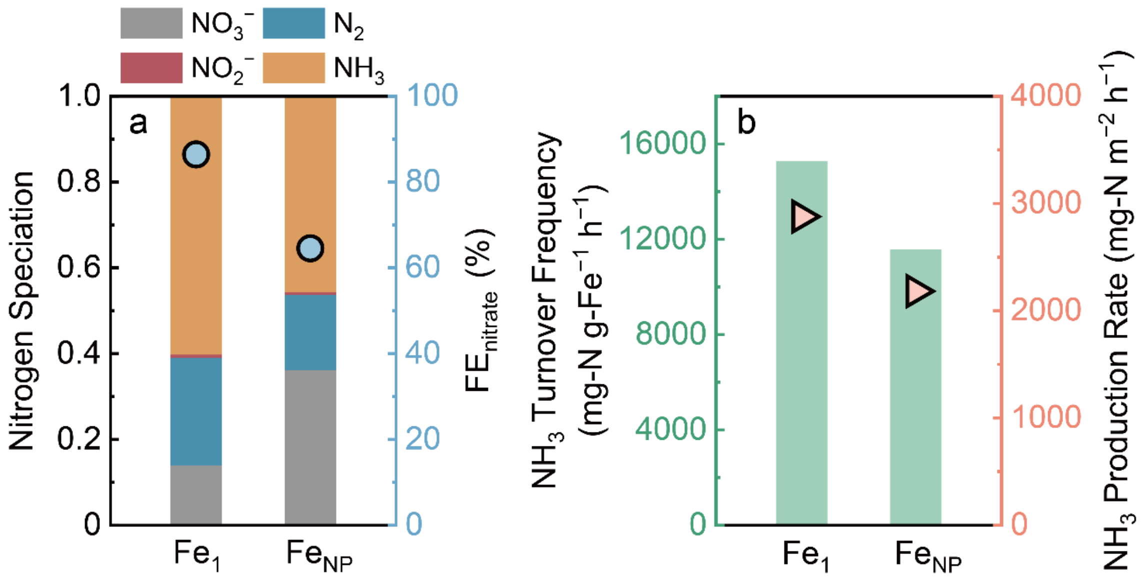

Fig. S9. Effect of applying Fe_1 or Fe_{NP} catalysts on (a) nitrogen speciation in the permeate (left axis) and $FE_{nitrate}$ (right axis) and (b) NH_3 turnover frequency (left axis) and NH_3 production rate (right axis).

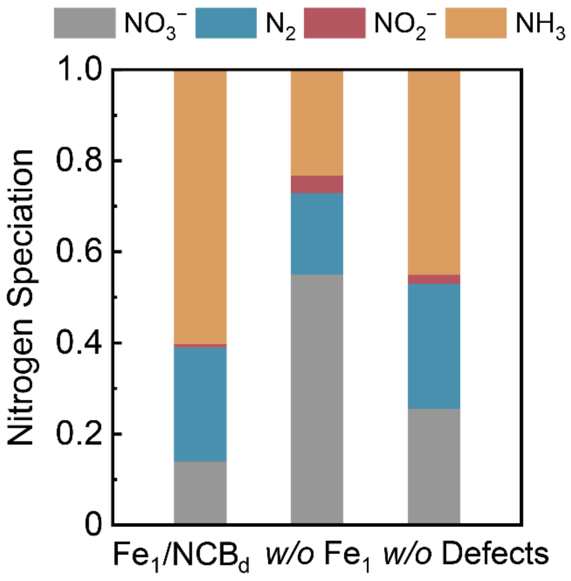

Fig. S10. Nitrogen speciation in the permeate using the Fe_1/NCB_d@CNT-FEM, a CNT-FEM incorporating defective N-doped carbon black without Fe_1 doping (i.e., NCB_d), and a CNT-EM containing N-doped carbon without the introduction of extra defects (i.e., Fe_1/NC).

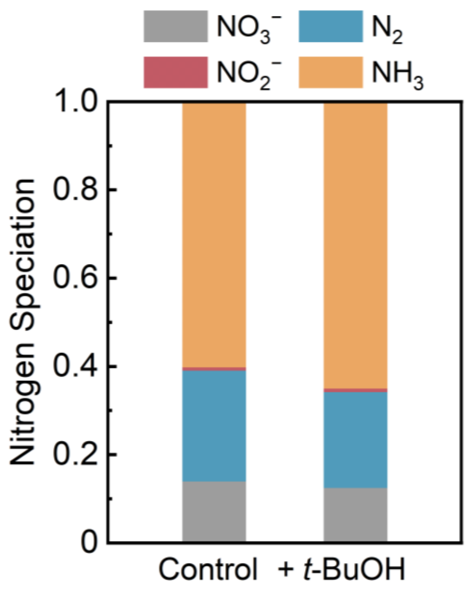

Fig. S11. Nitrogen speciation in the permeate (100 mg-N L^{-1} $NaNO_3$ in 50 mM Na_2SO_4) without or with *t*-BuOH (500 mM) in the feed solution as a radical quencher using the Fe_1/NCB_d@CNT-FEM.

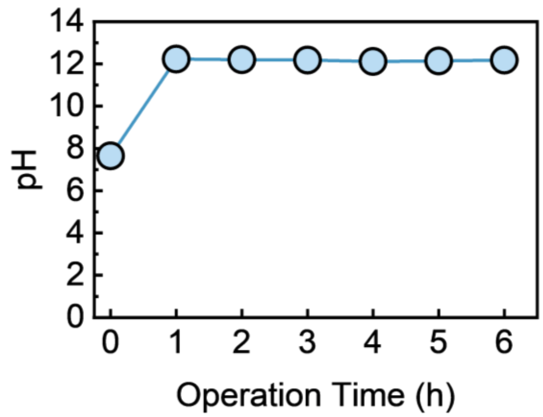

Fig. S12. Changes of pH in the permeate as a function of operation time.

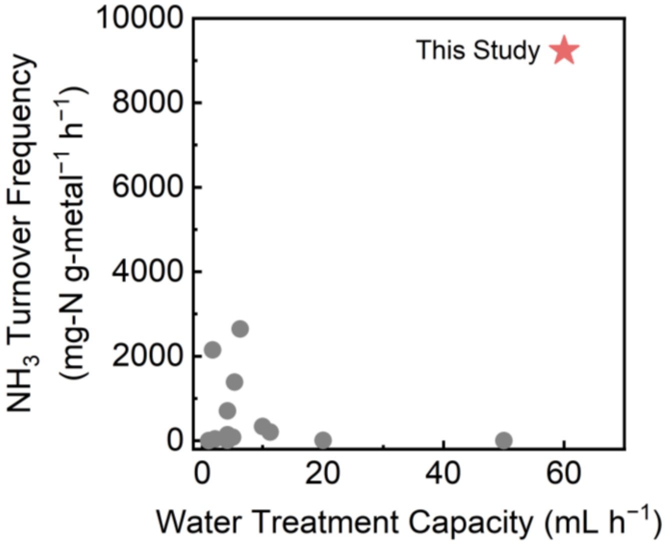

Fig. S13. Comparison of NH_3 turnover frequency and treatment capacity of nitrate-containing water for the Fe_1/NCB_d@CNT-FEM with other electrocatalysts reported in recent studies with initial NO_3^- concentrations ranging from 50 to 100 mg-N L^{-1} (data, see Table S3). The contribution of the NCB_d@CNT-FEM to NH_3 generation (see Fig. S10) is subtracted from the total NH_3 generation of the Fe_1/NCB_d@CNT-FEM when calculating the NH_3 turnover frequency of the Fe_1/NCB_d@CNT-FEM shown here ("This Study").

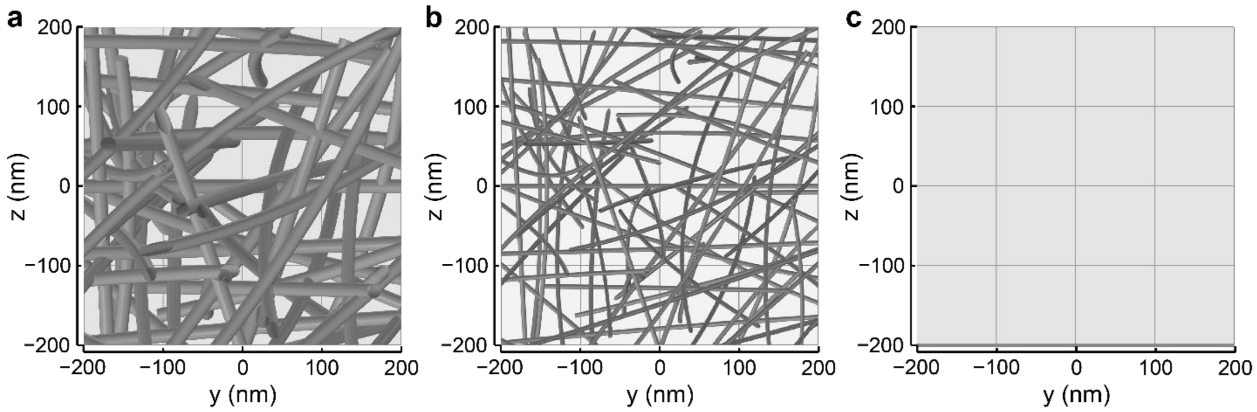

Fig. S14. CFD simulation models of (a) nanofiber interwoven framework with thick fibers (i.e., small membrane pores, (b) nanofiber interwoven framework with thin fibers (i.e., large membrane pores), and (c) flat plate control.

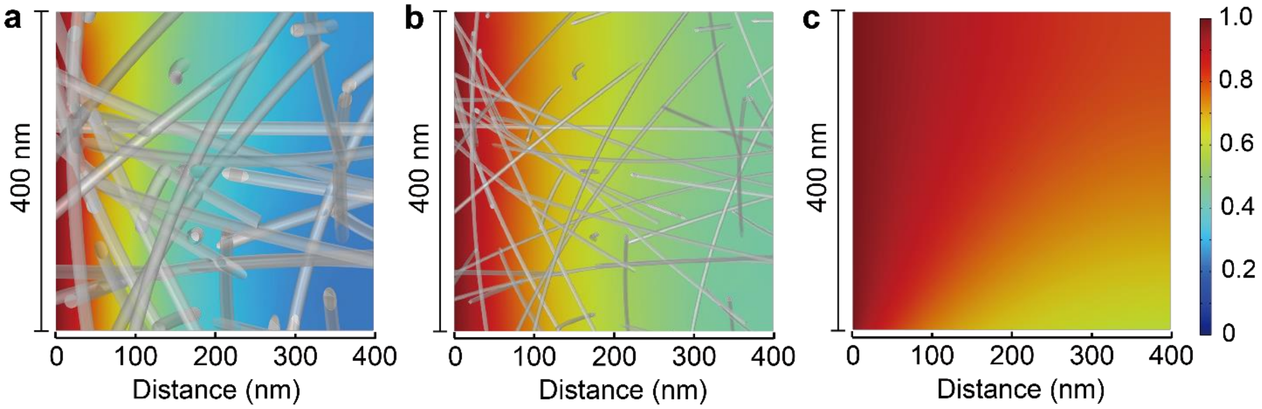

Fig. S15. CFD simulating the NO_3^- concentration distribution changes with flow distance of the interwoven frameworks with (a) thick fibers and (b) thin fibers and (c) the flat plate control.

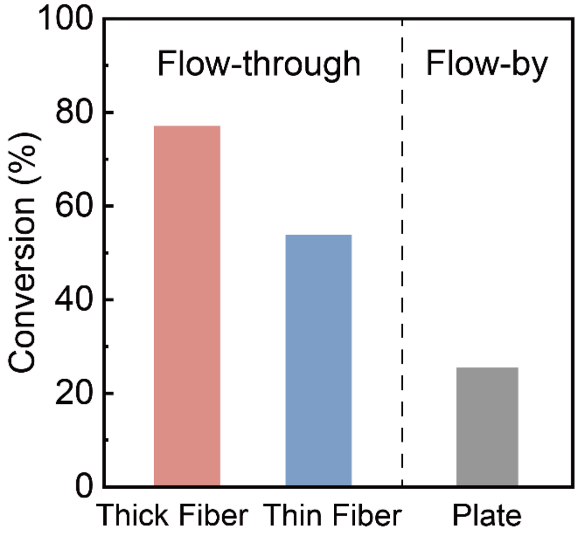

Fig. S16. CFD simulation showing the NO_3^- conversion under flow-through and flow-by modes with thick fiber, thin fiber, and flat plate conditions.

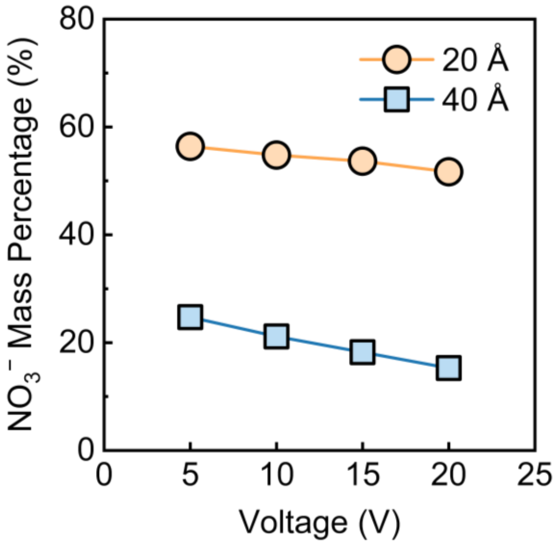

Fig. S17. MD simulation showing the changes of mass percentages of NO_3^- within 0.5 nm distance to the planes under different membrane pore sizes as a function of voltage.

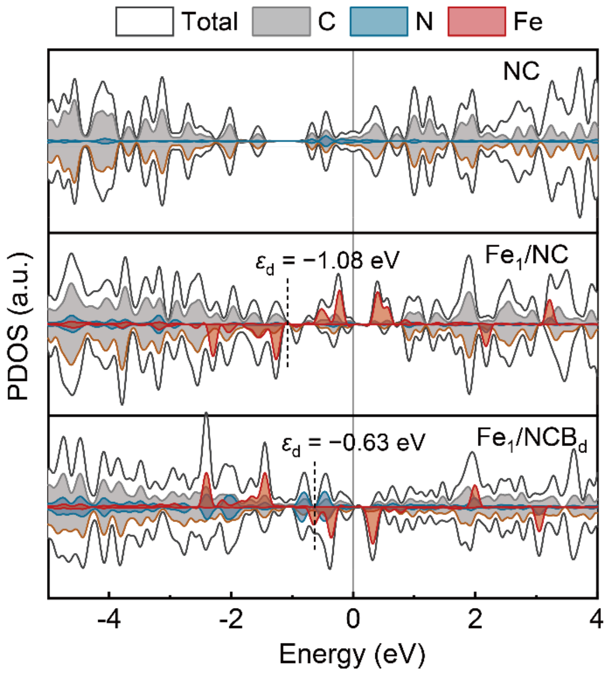

Fig. S18. Projected density of states (PDOS) of NO_3^- adsorbed on NC, Fe_1/NC, and Fe_1/NCB_d. The grey line represents the Fermi level. d-band centers (ε_d) of the catalysts are indicated by dashed lines.

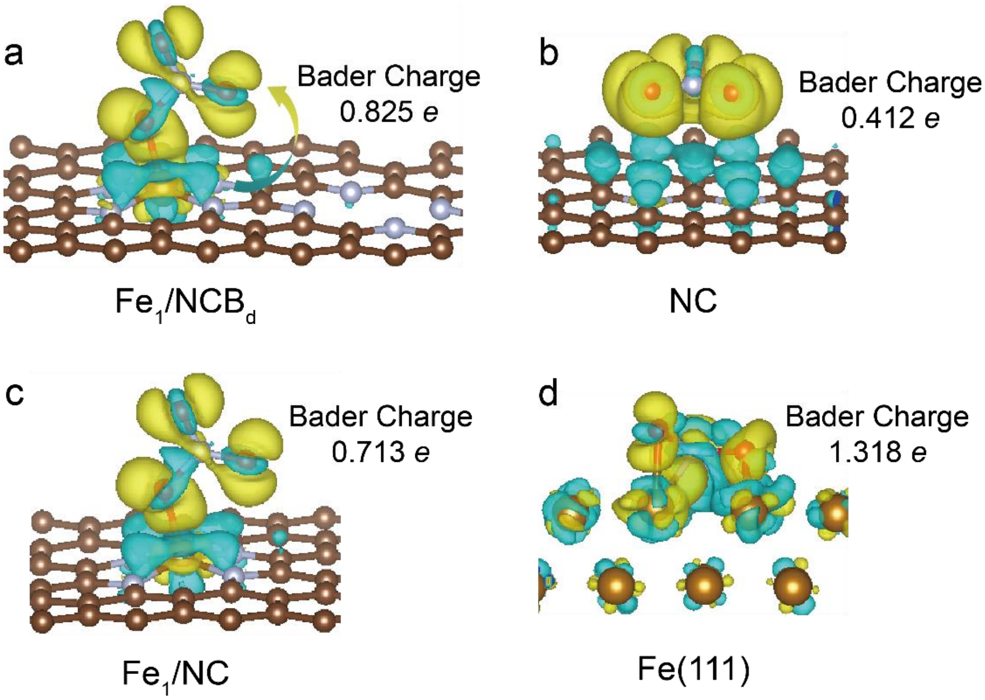

Fig. S19. Electron density differences of NO_3^- adsorbed on (a) Fe_1/NCB_d, (b) NC, (c) Fe_1/NC, and (d) Fe(111) surfaces and corresponding Bader charges. Areas of electron depletion are indicated by cyan, and areas of electron accumulation are indicated by yellow.

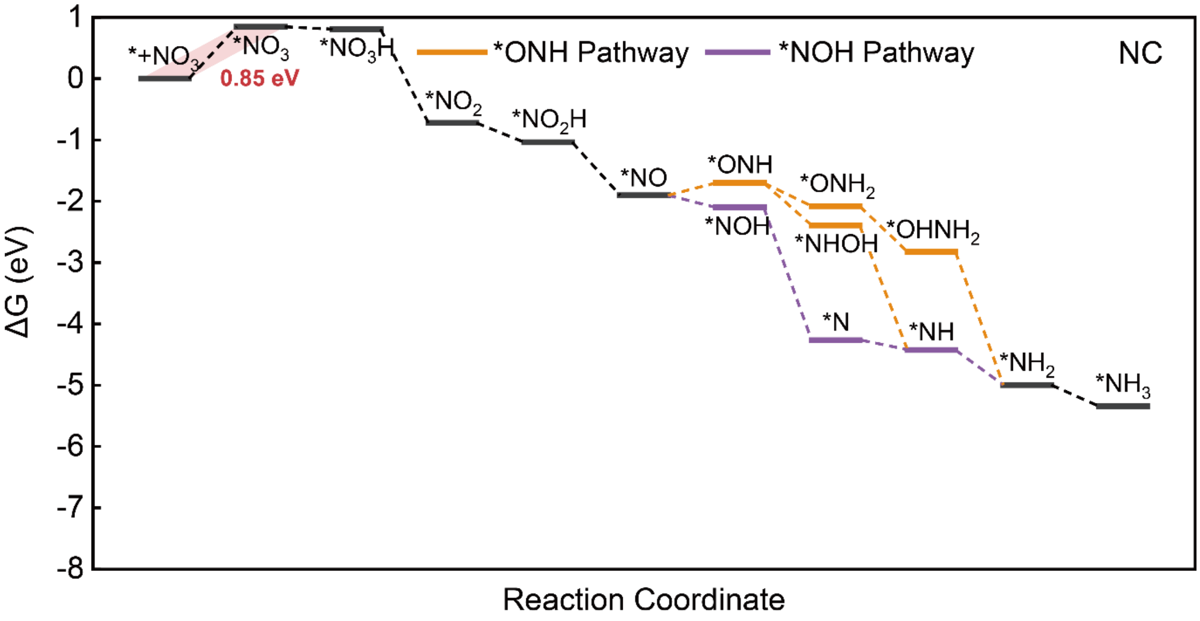

Fig. S20. Free energy diagrams of different reaction pathways for NO_3^- reduction to NH_3 on NC.

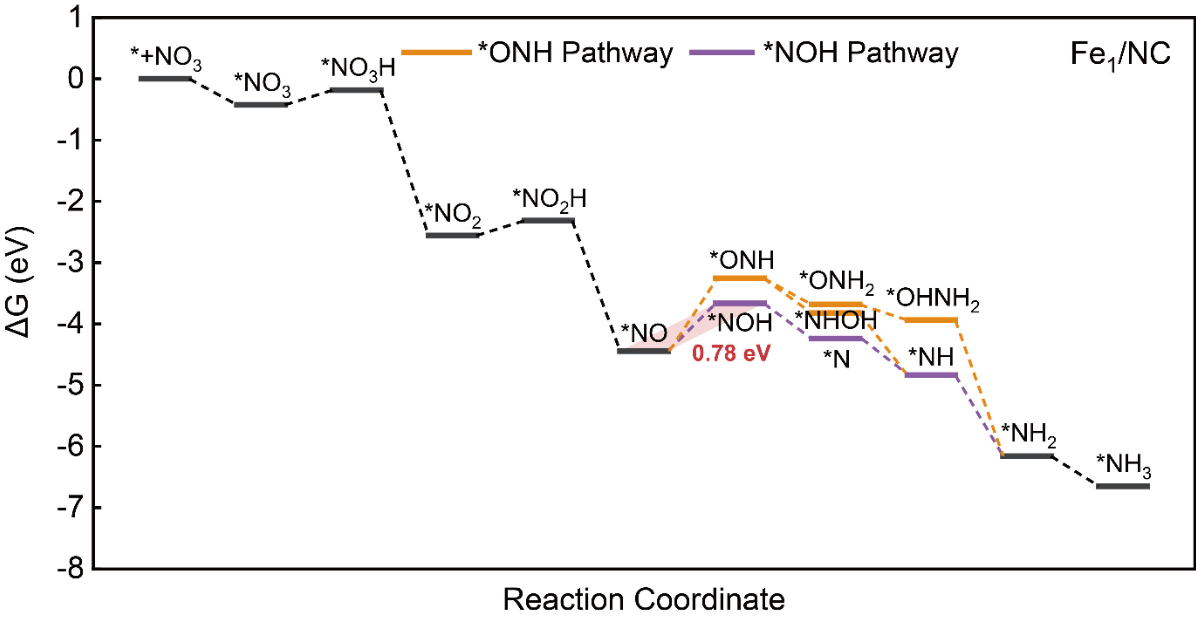

Fig. S21. Free energy diagrams of different reaction pathways for NO_3^- reduction to NH_3 on Fe_1/NC.

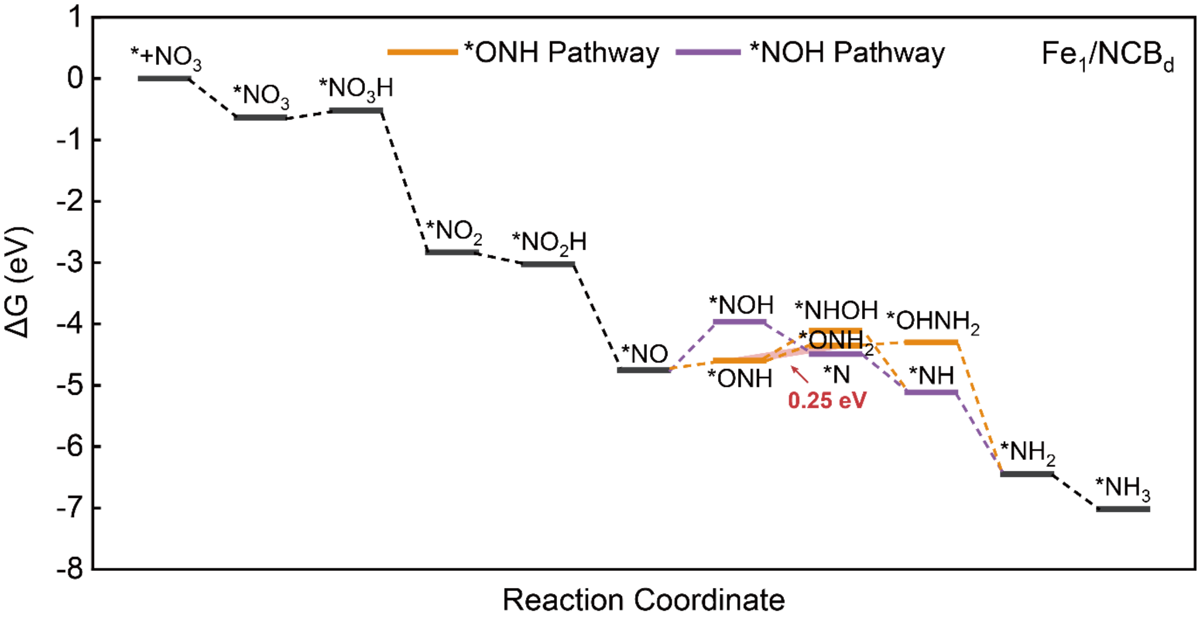

Fig. S22. Free energy diagrams of different reaction pathways for NO_3^- reduction to NH_3 on Fe_1/NCB_d.

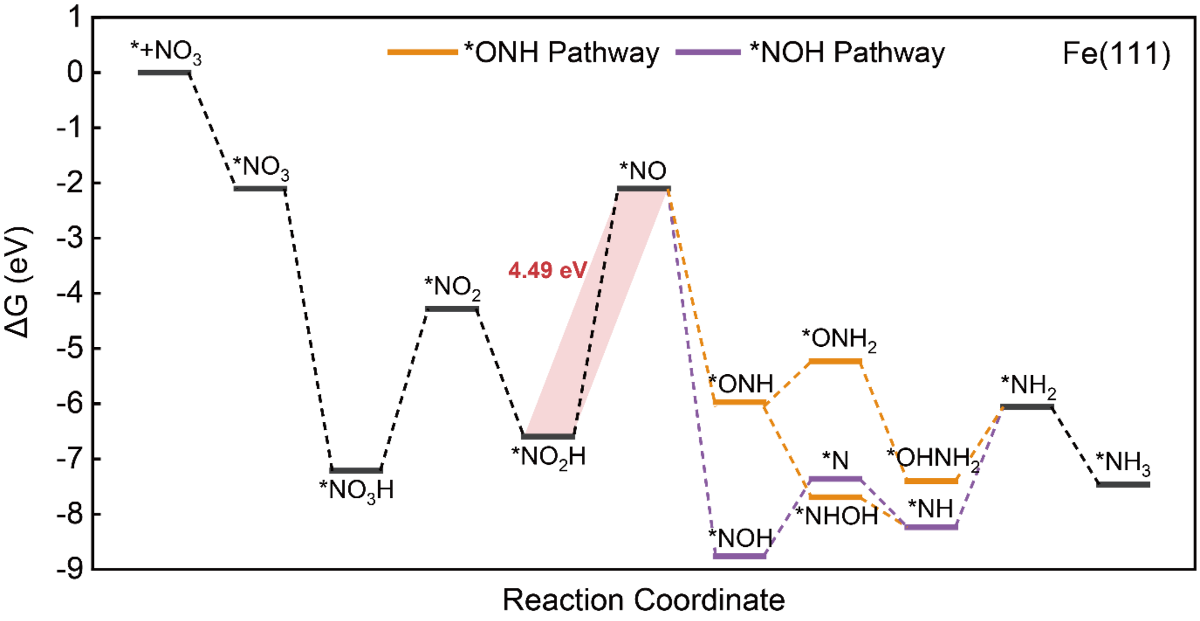

Fig. S23. Free energy diagrams of different reaction pathways for NO_3^- reduction to NH_3 on Fe(111).

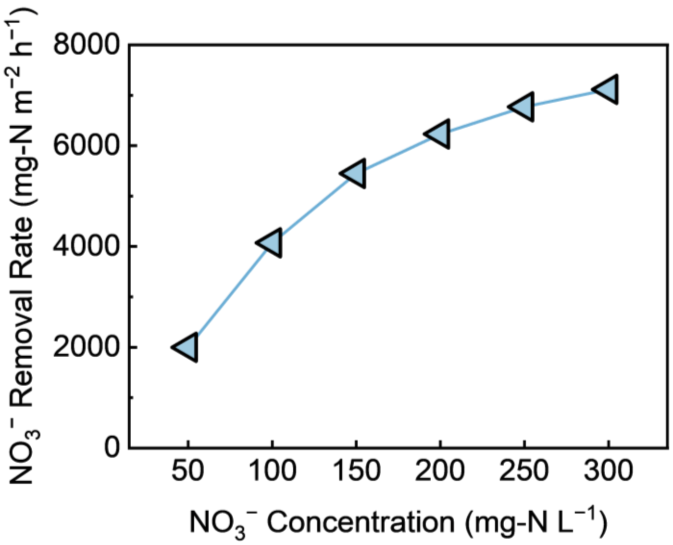

Fig. S24. Effect of initial NO_3^- concentration (50–300 mg-N L^{-1}) on NO_3^- removal rate using the Fe_1/NCB_d@CNT-FEM.

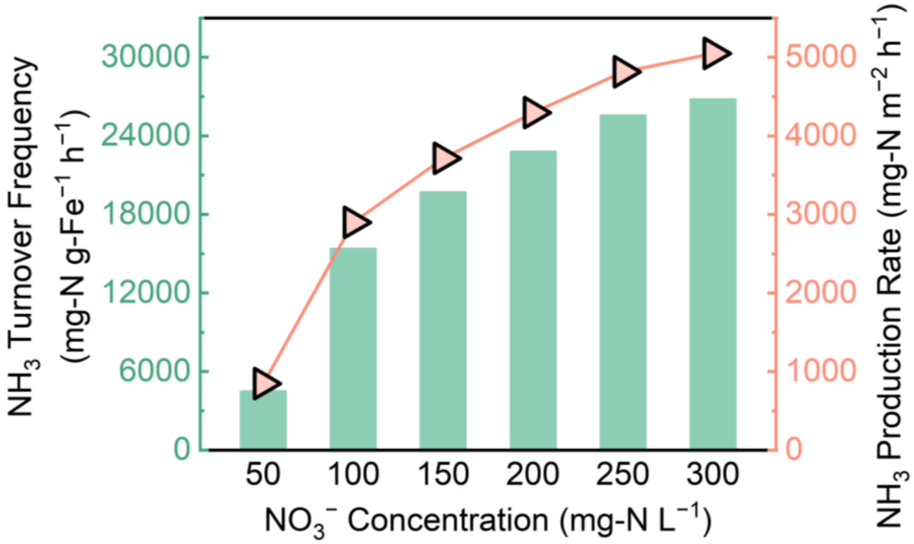

Fig. S25. Effect of initial NO_3^- concentration on NH_3 turnover frequency (left axis) and NH_3 production rate (right axis) using the Fe_1/NCB_d@CNT-FEM.

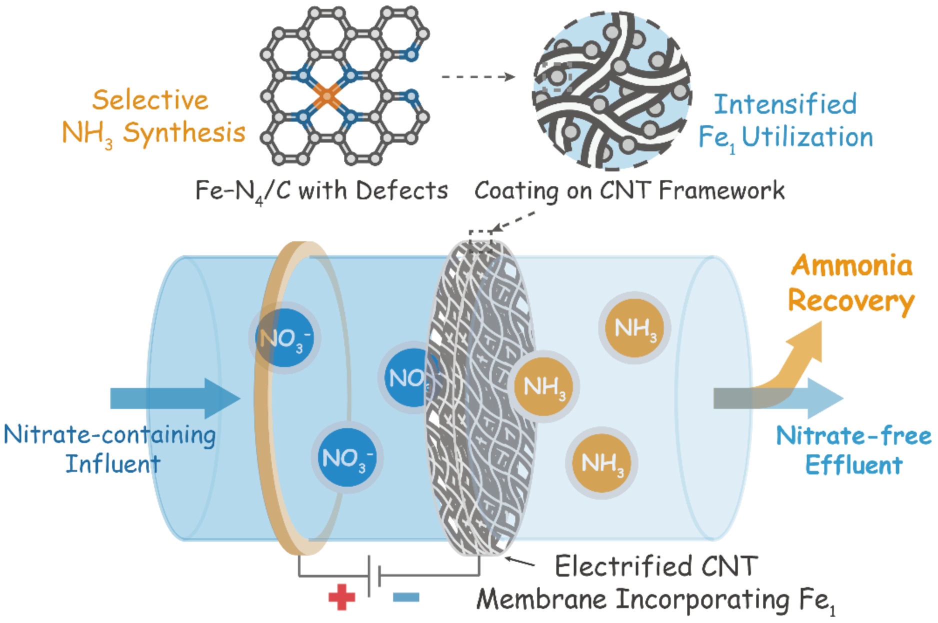

Fig. S26. Schematic illustrating the concept of intensifying atomic utilization efficiency under flow-through operation by applying a free-standing EM consisting of a CNT interwoven framework coated with Fe_1 catalysts to realize efficient nitrate removal and ammonia production.

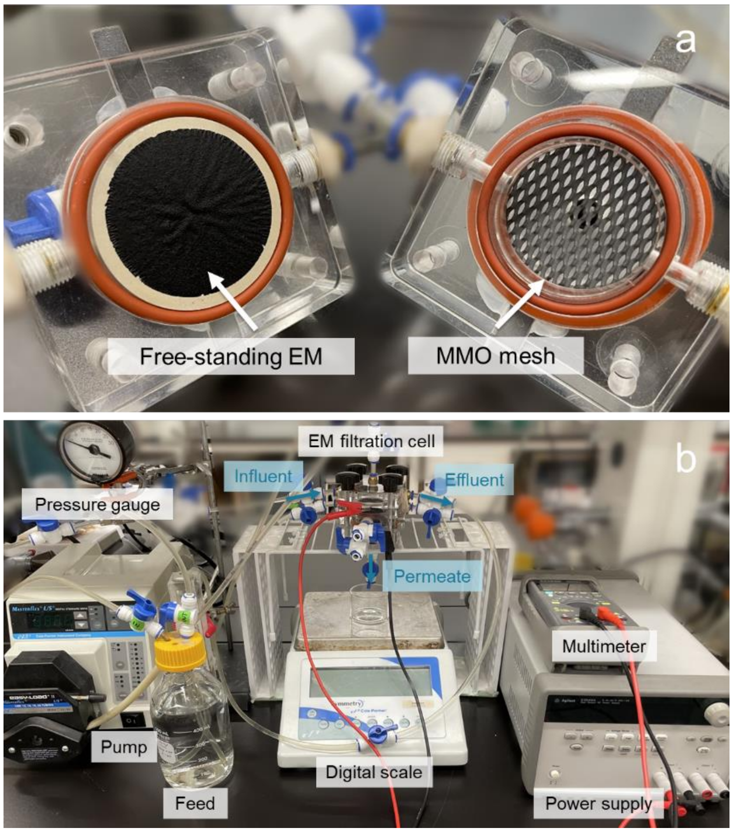

Fig. S27. Photographs of (a) electrified membrane filtration cell and (b) cross-flow electrofiltration system, reported previously.(*36*) A free-standing EM (geometric surface area of 12.6 cm^2) and a mixed metal oxide mesh electrode serve as the cathode and anode, respectively. Experiments were conducted at a cross-flow rate of 200 mL min^{-1} and a permeate flow rate of 1 mL min^{-1}.

Photo Credit: Xiaoxiong Wang; Shenzhen International Graduate School, Tsinghua University.

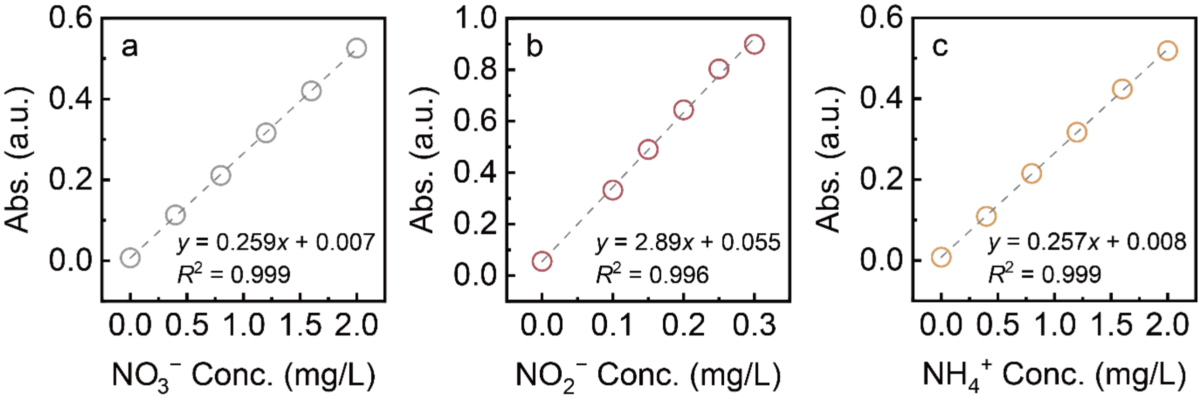

Fig. S28. Standard curves for colorimetric quantification of (a) NO_3^-, (b) NO_2^-, and (c) NH_4^+.

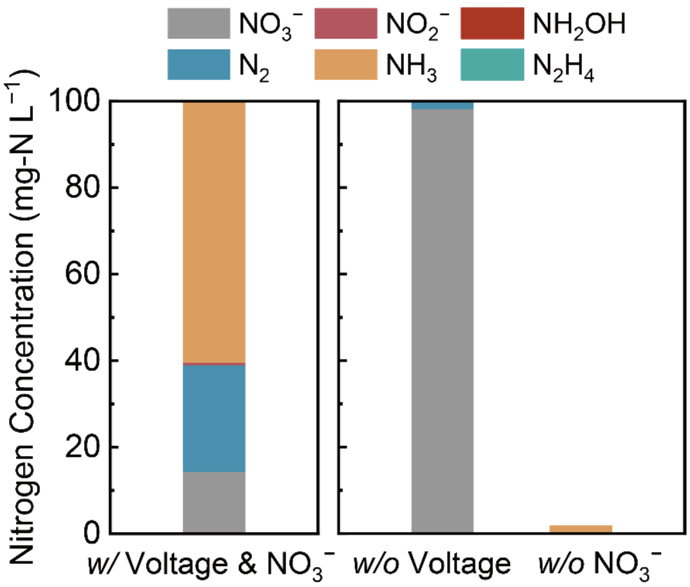

Fig. S29. Concentration of nitrogen species in the permeate when treating a feed solution (100 mg-N L^{-1} $NaNO_3$ in 50 mM Na_2SO_4) with (left panel) or without applied potential or a feed solution without NO_3^- (i.e., 50 mM Na_2SO_4) with potential (right panel) using the Fe_1/NCB_d@CNT-FEM.

The concentrations of NH_2OH and N_2H_4 were determined based on colorimetric methods using trichloroacetic acid, 8-quinolinol, and Na_2CO_3 solution and 4-dimethylaminobenzaldehyde and hydrochloric acid solution as the chromogenic agents at detecting wavelengths of 705 nm and 475 nm, respectively.

Table S1. Contents of Fe and N in Fe_1/NCB_d catalysts

Element	Composition	Binding energy (eV)	Area percent (%)
Fe	$2p_{3/2}$	711.7	36.3
	$2p_{3/2}$ sat	716.9	24.2
	$2p_{1/2}$	724.9	28.9
	$2p_{1/2}$ sat	730.6	10.6
N	Pyridinic-N	398.6	20.1
	Pyrrolic-N	399.9	43.7
	Graphitic-N	401.4	36.2

Table S2. Best-fit parameters extracted from the Fe *K*-edge FT EXAFS spectra

Sample	Shell	*CN*	*R* (Å)	σ^2 (Å^2)
Fe_1/NCB_d	Fe-N	3.7 ± 0.1	1.99	0.009
FePc	Fe-N	4	1.91	0.001
Fe foil	Fe-Fe	8	2.48	0.005

CN: coordination number, *R*: interatomic distance, σ^2: Debye-Waller factor.

Table S3. Comparison of nitrate reduction performance of Fe_1/NCB_d@CNT-FEM with that of reported electrocatalysts

Electrocatalyst	Initial NO_3^- (mg-N L^{-1})	Capacity (mL h^{-1})	NO_3^- removal rate (mg-N m^{-2} h^{-1})	NH_3 turnover frequency (mg-N g-metal^{-1} h^{-1})	Energy consumption per order (kWh m^{-3})	Ref.*
Fe_1/NCB_d@CNT-FEM	100	60	4109	**15059**	9.4	**This work**
CL-Fe@C	100	2	1865	41	11.3	Su et al. (*37*)
Fe(20%)@N-C	50	4	192	11	41.3	Duan et al. (*38*)
Co_3O_4-TiO_2/Ti	50	50	1766	3	9.7	Gao et al. (*39*)
FeNi/g-mesoC/NF	50	4	367	4	16.7	Chen et al. (*40*)
$Ni(OH)_2$@Ni	70	100	5548	18	9.4	Zheng et al. (*41*)
Cu_3P/CF	50	20	2443	4	29.3	Yao et al. (*42*)
Cu-NPC	50	5	659	1384	5.4	Zhao et al. (*43*)
Co-Fe@Fe_2O_3	50	5	1074	82	9.2	Zhang et al. (*44*)
Cu MNC	100	4	3746	702	4.6	Xue et al. (*45*)
Cu@AC	100	11	419	200	134.0	Sun et al. (*46*)
Fe@N_{10}-C	50	6	1099	2646	12.7	Zhang et al. (*47*)
Cu SAAs	100	4	682	139	20.8	Li et al. (*48*)
Fe/NFs	100	1	838	3	130.2	Zhang et al. (*49*)
FeSA/MXene	100	10	1336	338	12.0	Ren et al. (*50*)
Meso-Fe–N–C	100	2	268	2147	130.8	Fan et al. (*51*)

*Data selected for the analysis are collected from papers published in 2019−2023.

Table S4. Constituents of simulated brackish water (Nanotechnology-Enabled Water Treatment Engineering Research Center standard)

Constituents	Concentration (mg L^{-1})	Concentration (mM)
Bicarbonate (HCO_3^-)	244	4.0
Calcium (Ca^{2+})	120	3.0
Chloride (Cl^-)	1203	33.9
Magnesium (Mg^{2+})	49	2.0
Silica (SiO_2)	20	0.33
Nitrate (NO_3^-)*	443 (100.0 as N)	7.1
Sodium (Na^+)	750	39.7
Sulfate (SO_4^{2-})	194	2.0
Ionic strength		56
pH	8.0 ± 0.25	

*Nitrate concentration is modified to be consistent in this study.

REFERENCES AND NOTES

1. F. Cheng, K. Van Meter, D. Byrnes, N. Basu, Maximizing US nitrate removal through wetland protection and restoration. *Nature* **588**, 625–630 (2020).

2. C. Yu, X. Huang, H. Chen, H. C. J. Godfray, J. S. Wright, J. W. Hall, P. Gong, S. Ni, S. Qiao, G. Huang, Y. C. Xiao, J. Zhang, Z. Feng, X. T. Ju, P. Ciais, N. C. Stenseth, D. O. Hessen, Z. L. Sun, L. Yu, W. J. Cai, H. H. Fu, X. M. Huang, C. Zhang, H. B. Liu, J. Taylor, Managing nitrogen to restore water quality in China. *Nature* **567**, 516–520 (2019).

3. J. C. Ho, A. M. Michalak, N. Pahlevan, Widespread global increase in intense lake phytoplankton blooms since the 1980s. *Nature* **574**, 667–670 (2019).

4. B. R. Scanlon, S. Fakhreddine, R. C. Reedy, Q. Yang, J. G. Malito, Drivers of spatiotemporal variability in drinking water quality in the United States. *Environ. Sci. Technol.* **56**, 12965–12974 (2022).

5. US EPA, "National primary drinking water regulations" (2024); www.epa.gov/ground-water-and-drinking-water/national-primary-drinking-water-regulations.

6. EU Parliament, "Monitoring of nitrogen in water in the EU" (2022); www.europarl.europa.eu/RegData/etudes/STUD/2022/734713/IPOL_STU(2022)734713_EN.pdf.

7. A. Xu, Y. Wu, Z. Chen, G. Wu, Q. Wu, F. Ling, W. E. Huang, H. Hu, Towards the new era of wastewater treatment of China: Development history, current status, and future directions. *Water Cycle* **1**, 80–87 (2020).

8. T. M. Bowles, S. S. Atallah, E. E. Campbell, A. Gaudin, W. R. Wieder, A. S. Grandy, Addressing agricultural nitrogen losses in a changing climate. *Nat. Sustain.* **1**, 399–408 (2018).

9. B. Eickhout, A. F. Bouwman, H. van Zeijts, The role of nitrogen in world food production and environmental sustainability. *Agr Ecosyst Environ* **116**, 4–14 (2006).

10. Q. Gao, B. Yao, H. S. Pillai, W. Zang, X. Han, Y. Liu, S.-W. Yu, Z. Yan, B. Min, S. Zhang, Synthesis of core/shell nanocrystals with ordered intermetallic single-atom alloy layers for nitrate electroreduction to ammonia. *Nat. Synth.* 2, 624–634 (2023).

11. S. Han, H. Li, T. Li, F. Chen, R. Yang, Y. Yu, B. Zhang, Ultralow overpotential nitrate reduction to ammonia via a three-step relay mechanism. *Nat. Catal.* **6**, 402–414 (2023).

12. P. H. van Langevelde, I. Katsounaros, M. T. Koper, Electrocatalytic nitrate reduction for sustainable ammonia production. *Joule* **5**, 290–294 (2021).

13. K. Kim, A. Zagalskaya, J. L. Ng, J. Hong, V. Alexandrov, T. A. Pham, X. Su, Coupling nitrate capture with ammonia production through bifunctional redox-electrodes. *Nat. Commun.* **14**, 823 (2023).

14. J. Zhou, M. Wen, R. Huang, Q. Wu, Y. Luo, Y. Tian, G. Wei, Y. Fu, Regulating active hydrogen adsorbed on grain boundary defects of nano-nickel for boosting ammonia electrosynthesis from nitrate. *Energ. Environ. Sci.* **16**, 2611–2620 (2023).

15. M. Xie, S. Tang, Z. Li, M. Wang, Z. Jin, P. Li, X. Zhan, H. Zhou, G. Yu, Intermetallic single-atom alloy In–Pd bimetallene for neutral electrosynthesis of ammonia from nitrate. *J. Am. Chem. Soc.* **145**, 13957–13967 (2023).

16. H. Liu, J. Timoshenko, L. Bai, Q. Li, M. Rüscher, C. Sun, B. Roldan Cuenya, J. Luo, Low-coordination rhodium catalysts for an efficient electrochemical nitrate reduction to ammonia. *ACS Catal.* **13**, 1513–1521 (2023).

17. Y. Wang, M. Sun, J. Zhou, Y. Xiong, Q. Zhang, C. Ye, X. Wang, P. Lu, T. Feng, F. Hao, F. Liu, J. Wang, Y. Ma, J. Yin, S. Chu, L. Gu, B. Huang, Z. Fan, Atomic coordination environment engineering of bimetallic alloy nanostructures for efficient ammonia electrosynthesis from nitrate. *Proc. Natl. Acad. Sci. U.S.A.* **120**, e2306461120 (2023).

18. A. S. Fajardo, P. Westerhoff, C. M. Sanchez-Sanchez, S. Garcia-Segura, Earth-abundant elements a sustainable solution for electrocatalytic reduction of nitrate. *Appl. Catal. Environ.* **281**, 119465 (2021).

19. W. Guo, Z. Wang, X. Wang, Y. Wu, General design concept for single-atom catalysts toward heterogeneous catalysis. *Adv. Mater.* **33**, e2004287 (2021).

20. Z.-Y. Wu, M. Karamad, X. Yong, Q. Huang, D. A. Cullen, P. Zhu, C. Xia, Q. Xiao, M. Shakouri, F.-Y. Chen, J. Y. Kim, Y. Xia, K. Heck, Y. Hu, M. S. Wong, Q. Li, I. Gates, S. Siahrostami, H. Wang, Electrochemical ammonia synthesis via nitrate reduction on Fe single atom catalyst. *Nat. Commun.* **12**, 2870 (2021).

21. H. Luo, S. Li, Z. Wu, Y. Liu, W. Luo, W. Li, D. Zhang, J. Chen, J. Yang, Modulating the active hydrogen adsorption on Fe─N interface for boosted electrocatalytic nitrate reduction with ultra-long stability. *Adv. Mater.* **35**, e2304695 (2023).

22. Y. Jia, X. Yao, Defects in carbon-based materials for electrocatalysis: Synthesis, recognition, and advances. *Acc. Chem. Res.* **56**, 948–958 (2023).

23. Z. Qi, Y. Zhou, R. Guan, Y. Fu, J. B. Baek, Tuning the coordination environment of carbon-based single-atom catalysts via doping with multiple heteroatoms and their applications in electrocatalysis. *Adv. Mater.* **35**, e2210575 (2023).

24. A. Mehmood, M. Gong, F. Jaouen, A. Roy, A. Zitolo, A. Khan, M.-T. Sougrati, M. Primbs, A. M. Bonastre, D. Fongalland, G. Drazic, P. Strasser, A. Kucernak, High loading of single atomic iron sites in Fe–NC oxygen reduction catalysts for proton exchange membrane fuel cells. *Nature Catal.* **5**, 311–323 (2022).

25. C. Liu, G. Zhang, W. Zhang, Z. Gu, G. Zhu, Specifically adsorbed ferrous ions modulate interfacial affinity for high-rate ammonia electrosynthesis from nitrate in neutral media. *Proc. Natl. Acad. Sci. U.S.A.* **120**, e2209979120 (2023).

26. B. Wang, X. Zhu, X. Pei, W. Liu, Y. Leng, X. Yu, C. Wang, L. Hu, Q. Su, C. Wu, Y. Yao, Z. Lin, Z. Zou, Room-temperature laser planting of high-loading single-atom catalysts for high-efficiency electrocatalytic hydrogen evolution. *J. Am. Chem. Soc.* **145**, 13788–13795 (2023).

27. B. P. Chaplin, The prospect of electrochemical technologies advancing worldwide water treatment. *Acc. Chem. Res.* **52**, 596–604 (2019).

28. J. Wordsworth, T. M. Benedetti, S. V. Somerville, W. Schuhmann, R. D. Tilley, J. J. Gooding, The influence of nanoconfinement on electrocatalysis. *Angew. Chem. Int. Ed.* **61**, e202200755 (2022).

29. A. L. M. Reddy, A. Srivastava, S. R. Gowda, H. Gullapalli, M. Dubey, P. M. Ajayan, Synthesis of nitrogen-doped graphene films for lithium battery application. *ACS Nano* **4**, 6337–6342 (2010).

30. S. Kim, T. Ohta, G. Kwag, In situ structural investigation of iron phthalocyanine monolayer adsorbed on electrode surface by x-ray absorption fine structure. *Bull. Korean Chem. Soc* **21**, 589 (2000).

31. Y. Zhou, R. Duan, H. Li, M. Zhao, C. Ding, C. Li, Boosting electrocatalytic nitrate reduction to ammonia via promoting water dissociation. *ACS Catal.* **13**, 10846–10854 (2023).

32. H. Yang, L. Shang, Q. Zhang, R. Shi, G. I. Waterhouse, L. Gu, T. Zhang, A universal ligand mediated method for large scale synthesis of transition metal single atom catalysts. *Nat. Commun.* **10**, 4585 (2019).

33. C. Liu, J. Ma, M. Wang, J. Xu, C. Zhu, G. Zhu, Electrocatalytic nitrate reduction using iron single atoms for sustainable ammonium supplies to increase rice yield. *Proc. Natl. Acad. Sci. U.S.A.* **121**, e2408187121 (2024).

34. D. Hou, A. Iddya, X. Chen, M. Wang, W. Zhang, Y. Ding, D. Jassby, Z. J. Ren, Nickel-based membrane electrodes enable high-rate electrochemical ammonia recovery. *Environ. Sci. Technol.* **52**, 8930–8938 (2018).

35. J. Dai, Y. Tong, L. Zhao, Z. Hu, C.-T. Chen, C.-Y. Kuo, G. Zhan, J. Wang, X. Zou, Q. Zheng, W. Hou, R. Wang, K. Wang, R. Zhao, X. K. Gu, Y. Yao, L. Zhang, Spin polarized Fe1−Ti pairs for highly efficient electroreduction nitrate to ammonia. *Nat. Commun.* **15**, 88 (2024).

36. X. Wang, X. Wu, W. Ma, X. Zhou, S. Zhang, D. Huang, L. R. Winter, J.-H. Kim, M. Elimelech, Free-standing membrane incorporating single-atom catalysts for ultrafast electroreduction of low-concentration nitrate. *Proc. Natl. Acad. Sci. U.S.A.* **120**, e2217703120 (2023).

37. L. Su, D. Han, G. Zhu, H. Xu, W. Luo, L. Wang, W. Jiang, A. Dong, J. Yang, Tailoring the assembly of iron nanoparticles in carbon microspheres toward high-performance electrocatalytic denitrification. *Nano Lett.* **19**, 5423–5430 (2019).

38. W. Duan, G. Li, Z. Lei, T. Zhu, Y. Xue, C. Wei, C. Feng, Highly active and durable carbon electrocatalyst for nitrate reduction reaction. *Water Res.* **161**, 126–135 (2019).

39. J. Gao, B. Jiang, C. Ni, Y. Qi, Y. Zhang, N. Oturan, M. A. Oturan, Non-precious Co_3O_4-TiO_2/Ti cathode based electrocatalytic nitrate reduction: Preparation, performance and mechanism. *Appl. Catal. Environ.* **254**, 391–402 (2019).

40. X. Chen, T. Zhang, M. Kan, D. Song, J. Jia, Y. Zhao, X. Qian, Binderless and oxygen vacancies rich FeNi/graphitized mesoporous carbon/Ni foam for electrocatalytic reduction of nitrate. *Environ. Sci. Technol.* **54**, 13344–13353 (2020).

41. W. Zheng, L. Zhu, Z. Yan, Z. Lin, Z. Lei, Y. Zhang, H. Xu, Z. Dang, C. Wei, C. Feng, Self-activated Ni cathode for electrocatalytic nitrate reduction to ammonia: From fundamentals to scale-up for treatment of industrial wastewater. *Environ. Sci. Technol.* **55**, 13231–13243 (2021).

42. F. Yao, M. Jia, Q. Yang, F. Chen, Y. Zhong, S. Chen, L. He, Z. Pi, K. Hou, D. Wang, X. Li, Highly selective electrochemical nitrate reduction using copper phosphide self-supported copper foam electrode: Performance, mechanism, and application. *Water Res.* **193**, 116881 (2021).

43. X. Zhao, K. Zhao, X. Quan, S. Chen, H. Yu, Z. Zhang, J. Niu, S. Zhang, Efficient electrochemical nitrate removal on Cu and nitrogen doped carbon. *Chem. Eng. J.* **415**, 128958 (2021).

44. S. Zhang, M. Li, J. Li, Q. Song, X. Liu, High-ammonia selective metal-organic framework-derived Co-doped Fe/Fe_2O_3 catalysts for electrochemical nitrate reduction. *Proc. Natl. Acad. Sci. U.S.A.* **119**, e2115504119 (2022).

45. Y. Xue, Q. Yu, Q. Ma, Y. Chen, C. Zhang, W. Teng, J. Fan, W.-X. Zhang, Electrocatalytic hydrogenation boosts reduction of nitrate to ammonia over single-atom Cu with Cu(I)-N_3C_1 sites. *Environ. Sci. Technol.* **56**, 14797–14807 (2022).

46. J. Sun, S. Garg, J. Xie, C. Zhang, T. D. Waite, Electrochemical reduction of nitrate with simultaneous ammonia recovery using a flow cathode reactor. *Environ. Sci. Technol.* **56**, 17298–17309 (2022).

47. S. Zhang, M. Li, J. Li, Q. Song, X. Liu, N-doped carbon–iron heterointerfaces for boosted electrocatalytic active and selective ammonia production. *Proc. Natl. Acad. Sci. U.S.A.* **120**, e2207080119 (2023).

48. P. Li, L. Liao, Z. Fang, G. Su, Z. Jin, G. Yu, A multifunctional copper single-atom electrocatalyst aerogel for smart sensing and producing ammonia from nitrate. *Proc. Natl. Acad. Sci. U.S.A.* **120**, e2305489120 (2023).

49. F. Zhang, J. Luo, J. Chen, H. Luo, M. Jiang, C. Yang, H. Zhang, J. Chen, A. Dong, J. Yang, Interfacial assembly of nanocrystals on nanofibers with strong interaction for electrocatalytic nitrate reduction. *Angew. Chem. Int. Ed Engl.* **62**, e202310383 (2023).

50. Y. Ren, F. Tian, L. Jin, Y. Wang, J. Yang, S. You, Y. Liu, Fluidic MXene electrode functionalized with iron single atoms for selective electrocatalytic nitrate transformation to ammonia. *Environ. Sci. Technol.* **57**, 10458–10466 (2023).

51. J. Fan, Y. Chen, X. Chen, Z. Wu, W. Teng, W. X. Zhang, Atomically dispersed iron enables high-efficiency electrocatalytic conversion of nitrate to dinitrogen on a N-coordinated mesoporous carbon architecture. *Appl. Catal. Environ.* **320**, 121983 (2023).
